# Huntingtin is an RNA binding protein and participates in *NEAT1*-mediated paraspeckles

**DOI:** 10.1126/sciadv.ado5264

**Published:** 2024-07-19

**Authors:** Manisha Yadav, Rachel J. Harding, Tiantian Li, Xin Xu, Terence Gall-Duncan, Mahreen Khan, Costanza Ferrari Bardile, Glen L. Sequiera, Shili Duan, Renu Chandrasekaran, Anni Pan, Jiachuan Bu, Tomohiro Yamazaki, Tetsuro Hirose, Panagiotis Prinos, Lynette Tippett, Clinton Turner, Maurice A. Curtis, Richard L.M. Faull, Mahmoud A. Pouladi, Christopher E. Pearson, Housheng Hansen He, Cheryl H. Arrowsmith

**Affiliations:** ^1^ Structural Genomics Consortium, University of Toronto, Toronto, ON, Canada.; ^2^ Department of Medical Biophysics, University of Toronto, Toronto, ON, Canada.; ^3^ Princess Margaret Cancer Centre, University Health Network, Toronto, ON, Canada.; ^4^ Department of Pharmacology and Toxicology, University of Toronto, Toronto, ON, Canada.; ^5^ Genetics and Genome Biology, The Hospital for Sick Children, Toronto, ON, Canada.; ^6^ Department of Molecular Genetics, University of Toronto, Toronto, ON, Canada.; ^7^ Department of Medical Genetics, Centre for Molecular Medicine and Therapeutics, Djavad Mowafaghian Centre for Brain Health, Edwin S. H. Leong Centre for Healthy Aging, Faculty of Medicine, University of British Columbia, British Columbia Children’s Hospital Research Institute, Vancouver, BC, Canada.; ^8^ Graduate School of Frontier Biosciences, Osaka University, Suita, Japan.; ^9^ Institute for Open and Transdisciplinary Research Initiatives, Osaka University, Suita, Japan.; ^10^ School of Psychology, University of Auckland, Auckland, New Zealand.; ^11^ University Research Centre for Brain Research, University of Auckland, Auckland, New Zealand.; ^12^ Anatomical Pathology, Pathology and Laboratory Medicine, Auckland City Hospital, Auckland, New Zealand.; ^13^ Anatomy and Medical Imaging, University of Auckland, Auckland, New Zealand.

## Abstract

Huntingtin protein, mutated in Huntington’s disease, is implicated in nucleic acid–mediated processes, yet the evidence for direct huntingtin–nucleic acid interaction is limited. Here, we show wild-type and mutant huntingtin copurify with nucleic acids, primarily RNA, and interact directly with G-rich RNAs in in vitro assays. Huntingtin RNA-immunoprecipitation sequencing from patient-derived fibroblasts and neuronal progenitor cells expressing wild-type and mutant huntingtin revealed long noncoding RNA *NEAT1* as a significantly enriched transcript. Altered *NEAT1* levels were evident in Huntington’s disease cells and postmortem brain tissues, and huntingtin knockdown decreased *NEAT1* levels. Huntingtin colocalized with *NEAT1* in paraspeckles, and we identified a high-affinity RNA motif preferred by huntingtin. This study highlights *NEAT1* as a huntingtin interactor, demonstrating huntingtin’s involvement in RNA-mediated functions and paraspeckle regulation.

## Teaser

Huntingtin is an RNA binding protein that interacts with G-rich sequences, including those in the paraspeckle lncRNA *NEAT1*.

## INTRODUCTION

Huntington’s disease (HD) is a rare autosomal dominant neurodegenerative disorder with a wide range of motor, cognitive, and psychological symptoms (*1*, *2*). HD is caused by expansions of a naturally occurring CAG (cytosine-adenine-guanine) repeat tract (encoding polyglutamine) in the huntingtin (*HTT*) gene (*3*) with selective vulnerability of layer 5a corticostriatal neurons (*4*, *5*). The *HTT* gene has 5 to 35 CAG repeats in unaffected individuals, while mutant *HTT* (*mHTT*) contains ≥36 CAG repeats (*6*, *7*). CAG repeat length is the primary driver of the age of onset in HD with other genetic factors such as polymorphic variants within DNA repair genes and repeat tract purity, also influencing the age of onset and progression of the disease (*8*, *9*).

HTT is a 348-kDa Huntingtin, Elongation factor 3, protein phosphatase 2A, TOR1 (HEAT) repeat protein thought to scaffold protein-protein interactions (*10*, *11*). Among these, HAP40 is the only structurally and biophysically characterized protein interactor, and forms a stable heterodimer with both HTT and mHTT (*12*–*14*). Notably, the N-terminal HEAT domain of HTT has a positively charged solvent-exposed surface, postulated to act as a binding site for nucleic acids (*13*). Indirectly, colocalization experiments have implicated HTT in RNA transport (*15*, *16*) and HTT may interact with its own mRNA (*17*).

While HTT has been primarily studied as a cytoplasmic protein, localization and roles for HTT in nuclear functions are increasingly recognized. Kegel *et al*. (*18*) initially demonstrated in 2002 that the HTT protein exhibited diffuse staining within the nuclei of cultured cells and certain neurons. Phosphorylation of serine residues in the first 17 amino acids of HTT (N17) has been shown to facilitate the relocalization of the HTT protein to the nucleus (*19*) where it engages in diverse functions such as DNA damage repair (*10*) and transcription regulation (*20*). Although evidence for direct interaction of HTT with RNA is limited, there is substantial literature implicating HTT in gene expression and stress responses through mechanisms that could involve HTT-RNA interactions. Furthermore, many RNA binding proteins, like fused in sarcoma (FUS) consist of prion-like domains enriched in disordered regions with low sequence complexity, that are also implicated in the formation of liquid-liquid phase separation (LLPS) (*21*). Similar to this, exon 1 of HTT (HTTex1), comprising polyglutamine and proline-rich prion-like domain regions, is known to undergo LLPS and transition to form higher-order assemblies, both in vitro and in cells (*22*, *23*). Both wild-type (WT) and mutant HTTex1 associate with Ago2 in P-bodies, suggesting a potential role for HTT in RNA-mediated gene silencing (*24*). In addition, HTT functions in the nucleus and subnuclear speckles, contributing to transcription repression and RNA processing (*18*). During oxidative stress, HTT translocates to the nucleus, colocalizing with SC35^+^ nuclear speckles (*25*). Associations with Caprin-1 and G3BP1, stress granule markers, could suggest a role for HTT in regulating RNA processing and translation under stress (*26*, *27*). These findings suggest HTT’s involvement in LLPS with proteins and RNA, potentially affecting cellular RNA-dependent processes such as gene regulation and stress response.

To better understand HTT’s potential role in RNA-mediated processes, we used biophysical, biochemical, and cell-based assays to investigate the interaction of HTT and mHTT with RNA. We demonstrate a direct interaction between HTT and RNA, identifying *NEAT1* as a major HTT-binding RNA in cells, supporting a role for HTT in RNA-mediated stress responses involving *NEAT1*-mediated nuclear paraspeckles.

## RESULTS

### HTT-HAP40 interacts with RNA in vitro

We previously reported biophysical and structural studies of recombinant HTT (polyglutamine length of 23 or Q23) and expanded mHTT (Q54), as well as their HTT-HAP40 complexes, a biologically important proteoform of HTT and an obligate interaction partner of HTT (*13*, *28*, *29*). An intriguing observation was that both HTT and mHTT samples, extracted from either insect or mammalian cells via one-step affinity purification, copurified with large amounts of nucleic acid, and additional purification steps were required to yield highly pure (>98%) HTT protein (fig. S1A).

**Fig. 1.**
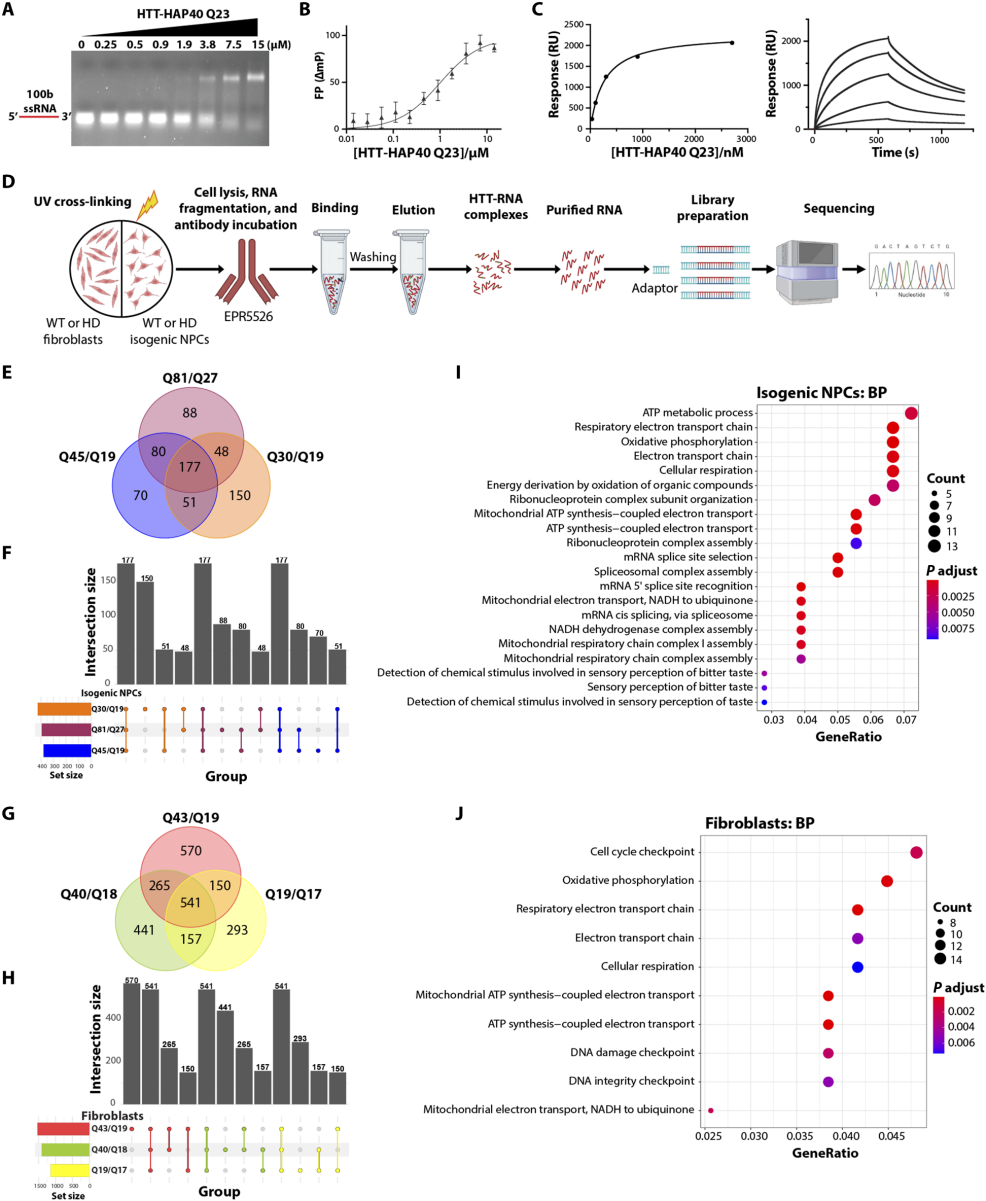
HTT protein binds RNA. (**A**) Representative EMSA images of increasing HTT-HAP40 Q23 protein (0 to 15 μM) binding with 1 μM of 100-mer random ssRNA. RNA is in red. EMSA, electrophoretic mobility shift assay. (**B**) Representative fluorescence polarization (FP) binding curve of HTT-HAP40 Q23 and 100-mer random ssRNA (*K*_d_ = 1 ± 0.6 μM). (**C**) Representative surface plasmon resonance binding curve and sensorgram of HTT-HAP40 Q23 and 100-mer random ssRNA (*K*_d_ = 0.4 ± 0.2 μM). (**D**) Schematic showing the protocol for HTT RNA-immunoprecipitation sequencing (RIP-seq) in isogenic neural progenitor cells (NPCs) and fibroblasts. UV, ultraviolet. (**E**) Venn diagram representing enriched RNA transcripts from immunoprecipitated (IP) samples of WT and HD NPCs. (**F**) Bar graph representing unique and overlapped enriched RNA transcripts from NPC IP samples. (**G**) Venn diagram representing enriched RNA transcripts from IP samples of WT and HD fibroblasts. (**H**) Bar graph representing unique and overlapping enriched RNA transcripts from fibroblasts IP samples. (**I** and **J**) Gene Ontology enrichment analysis for biological process (BP) in both WT and expanded isogenic NPCs (I) and fibroblasts (J). ATP, adenosine 5′-triphosphate; NADH, reduced form of nicotinamide adenine dinucleotide.

Using a 5-Carboxyfluorescein (5′-FAM)–labeled, single-stranded RNA (ssRNA) and 100–base pair (bp) double-stranded DNA (dsDNA) oligonucleotides with the same random sequence (table S1), we conducted electrophoretic mobility shift assays (EMSAs) with fully purified, recombinant HTT-HAP40 proteins (fig. S1A). We observed protein concentration–dependent band shifts indicating the formation of a complex of HTT-HAP40 with ssRNA, while dsDNA of the same sequence produced no shift (Fig. 1A and fig. S1B). Quantitative fluorescence polarization (FP) assays using the same oligonucleotide substrates confirmed a consistent trend, with HTT-HAP40 binding to ssRNA {*K*_d_ [dissociation constant (binding affinity)] = 1 ± 0.6 μM}, while no substantial binding to dsDNA (*K*_d_ > 15 μM, highest titration concentration) (Fig. 1B and fig. S1C). We further validated this finding using surface plasmon resonance analysis, yielding a similar trend with *K*_d_ values of 0.4 ± 0.2 μM and 2.0 ± 0.2 μM, for ssRNA and dsDNA, respectively (Fig. 1C and fig. S1D). Similar binding results were obtained for mHTT-HAP40 (Q54) compared to the WT protein for both ssRNA (*K*_d_ = 0.5 ± 0.3 μM) and dsDNA (*K*_d_ > 15 μM) oligonucleotide substrates. These findings suggest the limited influence of the polyglutamine tract on the observed interactions.

### HTT protein binds RNA and prefers G-rich RNA sequences

To gain insight into the endogenous RNAs bound by HTT in disease-relevant cells, we performed HTT RNA-immunoprecipitation sequencing (RIP-seq) in control and patient-derived fibroblast cells and isogenic neural progenitor cells (NPCs). Specifically, one WT (Q19/Q17) and two HD patient–derived fibroblast cell lines (Q40/Q18 and Q43/Q19) (*25*) and WT (Q30/Q19) and two isogenic HD NPC cell lines representing adult (Q45/Q19) and juvenile (Q81/Q27) disease-onset patients (*30*) were used. Cells were ultraviolet (UV)–cross-linked to form covalent bonds between RNA and protein, followed by immunoprecipitation (IP) of HTT using EPR5526 antibodies, purification of its associated RNA transcripts, and then sequencing of the prepared RNA libraries (Fig. 1D and fig. S2, A and B). The antibody EPR5526 used for RIP binds the HTT N17 domain (*31*); in HD patient brains, EPR5526 shows diffuse staining delineating pyramidal neurons in both control and HD cortices and HTT aggregates in HD cortices (*32*). Principle component analysis showed grouping distinctions between input and IP samples, as well as the separation of WT and HD samples in both NPCs and fibroblasts (fig. S2, C and D).

In total, 177 and 541 RNA transcripts were commonly and significantly enriched across all the WT and HD IP samples of NPCs (Fig. 1, E and F) and fibroblasts (Fig. 1, G and H), respectively. A subset of 53 transcripts were common between the isogenic NPCs and fibroblasts, including *NEAT1*, *ADARB1*, *EIF4A1*, *HOTAIRM1*, *BCL6*, *DOHH*, and *CHKB* as well as certain other *RP11* transcripts, micro-RNAs, and mitochondrial-encoded RNAs. We found significant enrichment of the HTT RNA transcript in WT NPCs, similar to a previously reported study (*17*); however, it was not enriched in any other samples. Gene Ontology enrichment analysis for biological processes and cellular components showed that the enriched transcripts were associated with pathways involved in mitochondrial adenosine 5′-triphosphate synthesis–coupled electron transport, RNA splicing, spliceosome machinery, 5′ splice site recognition, and several other mitochondrial functional pathways such as oxidative phosphorylation, electron transport chain, and cellular respiration (Fig. 1, I and J, and fig. S3, A and B).

**Fig. 2.**
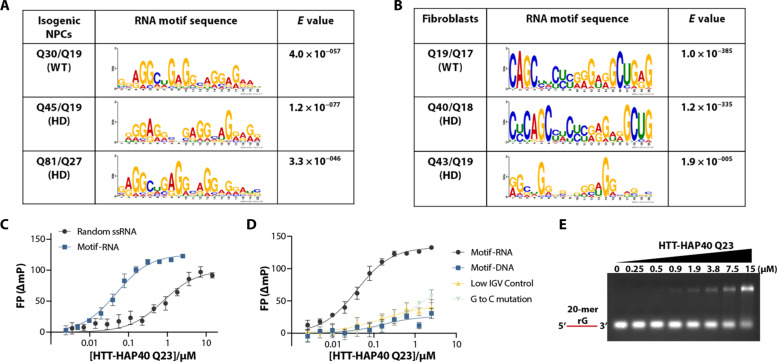
HTT protein prefers G-rich RNA sequences. (**A** and **B**) MEME identified HTT protein binding RNA motif sequences captured by isogenic NPCs (A) and fibroblasts (B) RIP-seq. (**C**) Representative FP binding curve of HTT-HAP40 Q23 and 100-mer random ssRNA (*K*_d_ = 1 ± 0.6 μM) and motif RNA (*K*_d_ = 40 ± 8.5 nM). (**D**) Representative FP binding curve of HTT-HAP40 Q23 and motif RNA, DNA form of motif RNA (*K*_d_ > 15 μM), low Integrative Genomic Viewer (IGV; *K*_d_ > 15 μM), and G to C–mutated RNA (*K*_d_ > 15 μM). NC, not calculated, outside of the range of protein concentrations tested. (**E**) Representative EMSA image of increasing HTT-HAP40 Q23 protein (0 to 15 μM) binding with 1 μM of 20-mer rG. RNA is in red.

To delve deeper into HTT-RNA interactions, we sought to determine whether HTT exhibits a preference for binding to specific RNA sequence(s). Multiple Expectation maximizations for Motif Elicitation (MEME) motif sequence analysis (*33*) identified a G-rich sequence HTT-binding RNA motif (GGAAGGCGAGGC) in both NPCs (Fig. 2A) and fibroblasts (Fig. 2B). To evaluate direct binding of HTT to the identified G-rich RNA motif, we used synthetic, 5′-FAM–labeled, 25-mer oligonucleotides bearing the identified RNA motif sequence in an FP assay where HTT proteins were titrated (table S1). HTT-HAP40 Q23 exhibited binding to the identified RNA motif (labeled as Motif-RNA) with a *K*_d_ value of 40 ± 8.5 nM compared to the same 100b random ssRNA used above (Fig. 1, A to C) with a *K*_d_ value of 1 ± 0.6 μM (Fig. 2C). We also assessed the binding affinity of HTT-HAP40 Q23 to the RNA motif sequence within a DNA backbone (labeled as Motif-DNA) which indicated a much weaker binding affinity (*K*_d_ = not calculated), supporting HTT’s preference for RNA over DNA for this motif (Fig. 2D). We further tested a region of *NEAT1* that had no peaks enriched in our RIP-seq data (labeled as Low IGV Control), which also showed weak binding to HTT-HAP40 Q23 (Fig. 2D).

Considering that the preferred RNA motifs were all G-rich, a sequence that is prone to secondary structures, we conducted circular dichroism spectroscopy analysis of the motifs (table S1) to assess for potential secondary structures (*34*). The spectral profiles for the motif sequence with either an RNA or DNA backbone indicated that they were probably forming G-quadruplexes (fig. S4A), which could indicate that HTT has a preference to bind to the RNA form of these structures. To test whether HTT’s binding is influenced by G-quadruplex structured elements, we replaced three guanines (G’s) in the RNA motif with cytosines [(G**C**AAG**C**CGAG**C**C)] (labeled as G to C mutation), which would disrupt the ability of the sequence to form a G-quadruplex (table S1). This G to C mutation resulted in significantly reduced binding of HTT-HAP40 Q23 (*K*_d_ = greater than the highest concentration tested; Fig. 2D).

To validate this finding, we conducted an EMSA assay using 20-mer ssRNA substrates (rG, rU, rC, and rA; table S1) with increasing concentrations of HTT-HAP40 Q23 protein. Our EMSA results revealed the formation of RNA-protein complexes in the presence of rG (Fig. 2E). However, no notable RNA-protein complex formation was observed in the presence of rU, rC, and rA substrates (fig. S4, B to D). Thus, our study suggests that HTT-HAP40 Q23 protein exhibits a preference for binding G-rich RNA motifs, aligning with our motif analysis and FP assay results. These findings contribute to our understanding of the molecular interactions involving HTT-HAP40 Q23 and its RNAs, particularly those with G-quadruplex forming sequences.

### Long noncoding RNA *NEAT1* is a highly enriched HTT-bound transcript

**Fig. 3.**
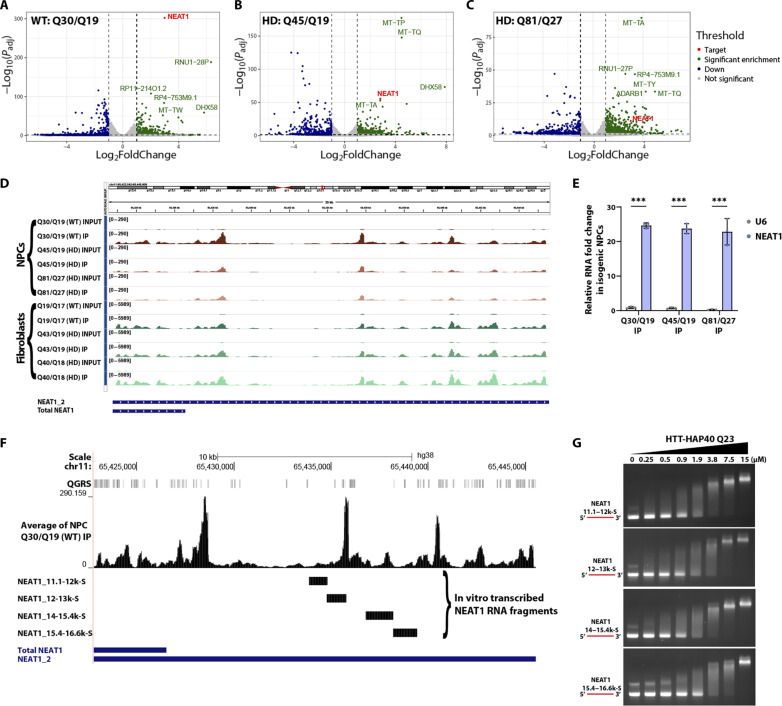
LncRNA *NEAT1* as an HTT-binding substrate. (**A** to **C**) Volcano plots showing lncRNA *NEAT1* as a significantly enriched target in the WT and expanded isogenic NPC IP samples (Log_2_ fold change cutoff: 1; *P* value cutoff: 0.05). (**D**) IGV snapshot showing the enrichment profile of *NEAT1* across individual NPC and fibroblast IPs after autoscaling to their respective Inputs. (**E**) Reverse transcription quantitative polymerase chain reaction (RT-qPCR) validation of *NEAT1* transcript enrichment in WT and expanded NPC IP samples compared to U6 control gene. Data were analyzed using two-way analysis of variance (ANOVA) and shown as means ± SD; *n* = 3 (technical replicates). ***P < 0.001. (**F**) Isogenic NPCs Q30/Q19 IP data mapped to *NEAT1*. Quadruplex-forming G-rich sequences (QGRS) mapped to *NEAT1* are shown as tall bars (in gray) across the top. In vitro transcribed *NEAT1* fragments (black bars underneath enriched peaks) are mapped to the *NEAT1* gene (in blue). (**G**) Representative EMSA images of increasing HTT-HAP40 Q23 protein (0 to 15 μM) binding with 0.25 µM of in vitro transcribed *NEAT1* RNA fragments. RNA is in red.

The long noncoding RNA (lncRNA) *NEAT1* was consistently the topmost significantly enriched transcript across all IP samples from different genetic backgrounds (Fig. 3, A to C, and fig. S5, A to C). Previous studies have highlighted the presence of abundant and conserved G-quadruplex motifs in *NEAT1*, with RNA binding proteins such as non-POU domain-containing octamer-binding (NONO) exhibiting specificity for these motifs (*35*). *NEAT1* displayed a consistent enrichment pattern across all RIP-seq samples (Fig. 3D). We used reverse transcription quantitative polymerase chain reaction (RT-qPCR) to quantify the enrichment of the *NEAT1* transcript in the IP fractions compared to the cytoplasmic RNA encoding the 40*S* ribosomal protein RPS28, as well as U6 and *MALAT1*, which are exclusively nuclear transcripts (Table 1). A robust signal was detected for *NEAT1* transcripts in the IP fractions compared to the housekeeping genes RPS28, U6, and lncRNA *MALAT1*, used as a negative control (Fig. 3E and fig. S5, D and E).

**Table 1.** Primer sequences used for RT-qPCR. List of forward and reverse primer sequences used in this study.

Gene	Primer sequence
RPS28f	CGATCCATCATCCGCAATG
RPS28r	AGCCAAGCTCAGCGCAAC
U6f	GAGAAGATTAGCATGGCCC
U6r	AATATGGAACGCTTCACGA
GAPDHf	CATGAGAAGTATGACAACAGCCT
GAPDHr	AGTCCTTCCACGATACCAAAGT
Total NEAT1f	CCAGTTTTCCGAGAACCAAA
Total NEAT1r	ATGCTGATCTGCTGCGTATG
NEAT1_2f	CTAGAGGCTCGCATTGTGTG
NEAT1_2r	GCCCACACGAAACCTTACAT
MALAT1f	TGGTGATGAAGGTAGCAGGC
MALAT1r	ATTGCCGACCTCACGGATTT

Given that the HTT-binding RIP-seq motif featured G-rich sequences (Fig. 2, A and B), and our recombinant HTT-HAP40 preferred G-rich ssRNA and G-quadruplexes (Fig. 2, C to E), we searched the *NEAT1* RIP-seq data for G-rich sequences. Using the QGRS mapper tool (https://bioinformatics.ramapo.edu/QGRS/index.php), employing parameters similar to a previous study (*35*), we identified putative G-quadruplex–forming sequences in *NEAT1_2*. Analyzing *NEAT1* enrichment profiles in NPC IP samples and QGRS data on the UCSC genome browser, we observed enrichment of HTT at multiple regions across *NEAT1*. Notably, these regions appeared to largely overlap with G-quadruplex–forming segments (Fig. 3F). To further explore the interactions between HTT and *NEAT1* RNA, we synthesized four in vitro transcribed sense RNA fragments of *NEAT1* (Fig. 3F), following the previously described protocol (*36*). EMSA of the resultant transcripts revealed that band shifts were observed in a concentration-dependent manner with increasing concentration of HTT-HAP40 Q23 protein, providing further evidence of a direct interaction between HTT protein and lncRNA *NEAT1* (Fig. 3G).

### *NEAT1* levels in NPCs, patient-derived fibroblasts, and human postmortem brain tissues are altered in HD

*NEAT1* levels are reported to be dysregulated in various neurodegenerative diseases, including HD (*37*–*40*). Nevertheless, the association between *NEAT1* and HD pathology remains unclear, with conflicting reports indicating either an increase or decrease in *NEAT1* expression levels in various HD models (*40*, *41*).

**Fig. 4.**
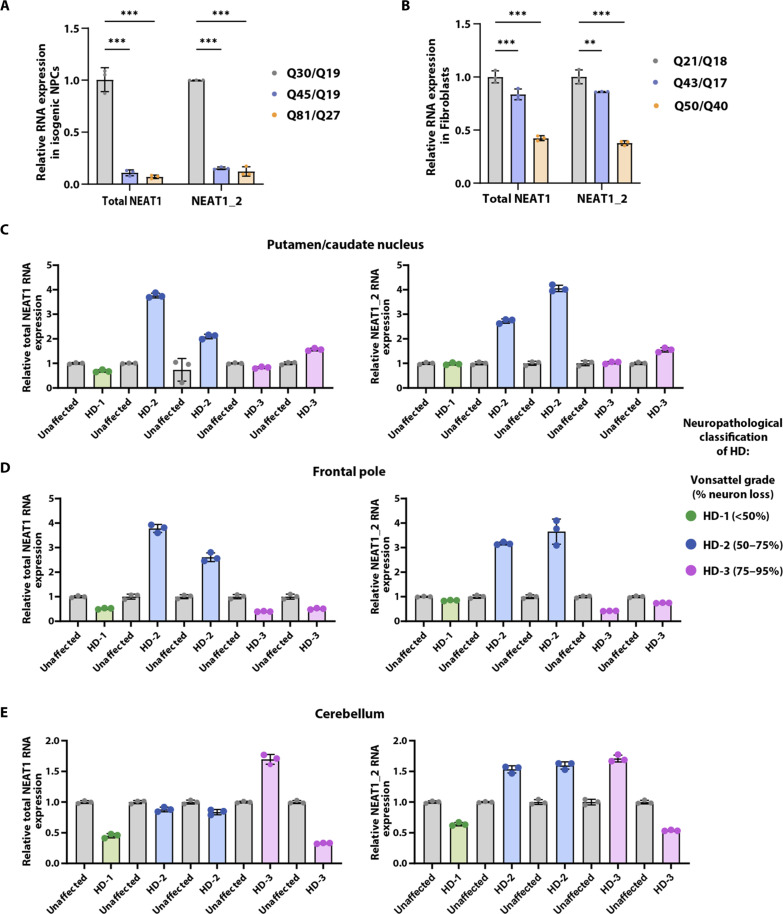
Altered levels of lncRNA *NEAT1* across WT and expanded cell lines and HD human brain samples. (**A** and **B**) RT-qPCR quantification of expression levels of total *NEAT1* and long *NEAT1* (*NEAT1_2*) isoform in WT and expanded isogenic NPCs (A) and patient-derived fibroblasts (B). U6 was used as the control gene and data were analyzed using two-way ANOVA. Data are shown as means ± SD; *n* = 3 (technical replicates). ***P < 0.001 and **P < 0.01. (**C** to **E**) RT-qPCR quantification of total *NEAT1* and long *NEAT1* (*NEAT1_2*) expression levels in putamen/caudate nucleus (C), frontal pole (D), and cerebellum (E) regions of human unaffected and HD patient brain (at different HD-grades). Age- and sex-matched unaffected brain tissues for each region were used to normalize data (gray). *N* = 5 HD (i.e., one individual from stage 1 HD, two individuals from stage 2, and two individuals from stage 3; total = 5 individuals) and *N* = 5 unaffected individuals per group per tissue, dots on bars represent three technical replicates per person. Green, blue, and purple colors of the dots indicate striatal neuropathological grade (HD-1 to HD-3); gray dots indicate unaffected controls. U6 was used as a control gene. See fig. S6 for an alternative figure of the same data. No statistics were applied because of the limited patient sample size.

The *NEAT1* locus produces two isoforms: *NEAT1_1* (short, ∼3.7 kb) and *NEAT1_2* (long, ∼22.7 kb) (Fig. 3D), with *NEAT1_2* being an essential component of paraspeckles (*36*, *42*). We assessed the levels of *NEAT1* in the isogenic NPC allelic series, with RT-qPCR using *NEAT1* isoform–specific primers (Table 1). Because the *NEAT1_1* sequence is identical to the 5′ sequence of the longer *NEAT1_2*, it is difficult to distinguish between the two (*43*). Therefore, primers that amplify the 5′ region of the gene will report on both *NEAT1_1* and *NEAT1_2* and such results are referred to as total *NEAT1* throughout the text, whereas *NEAT1_2* represents the long isoform (see also Fig. 3, D and F, and Table 1). The qPCR results indicated that both total *NEAT1* and *NEAT1_2* levels were significantly lower in the polyglutamine-expanded NPCs compared to WT (Fig. 4A). Similarly, qPCR of WT and HD fibroblast cell lines showed a significant reduction of *NEAT1* in the HD lines compared to WT (Q21/Q18), with the homozygous HD line displaying significantly lower *NEAT1* levels compared to the heterozygous HD cell lines (Fig. 4B).

To further investigate this finding, we mined publicly accessible RNA-seq datasets from both WT and HD cell and animal models. We found decreased *NEAT1* expression levels in the striatum of HD Q175 knock-in mice (B6J strain) compared to WT mice. In the Hdh knock-in mouse allelic series Hdh^Q50^, Hdh^Q92^, and Hdh^Q140^, the *NEAT1* transcript count decreased in the later stages (10 months) compared to WT and Hdh^Q20^ mice (*44*). These data align well with the cell line data in Fig. 4 (A and B), demonstrating lower *NEAT1* levels in HD cells and models, especially in the later stages of HD.

Subsequently, we conducted qPCR using cDNA from three distinct brain regions (putamen/caudate nucleus, frontal pole, and cerebellum) derived from postmortem brain tissues of patients with HD kindly provided by the Neurological Foundation Human Brain Bank in the Centre for Brain Research, University of Auckland with full consent from the donor families. These tissues were collected from patients with HD diagnosed with different grades of disease (table S2). To normalize the RT-qPCR data, we used age- and sex-matched unaffected brain tissues (table S2). The qPCR results suggest a potential relationship between *NEAT1* and human tissues from different HD grades, where we observed lower levels of both total *NEAT1* and *NEAT1_2* in HD grade 1, in all three brain regions. The *NEAT1* levels seem to increase as the disease progresses from grade 1 to grade 2, especially the putamen and frontal pole (Fig. 4, C to E, and fig. S6). This might potentially reflect the heightened stress on neurons. However, *NEAT1* levels appear to subsequently decrease in grade 3, which could possibly be attributed to either neuronal loss or the presence of mHTT (Fig. 4, C to E, and fig. S6). Larger sample sizes from different brain regions of unaffected and patients with HD across different grades are required for robust statistical analysis and to draw any conclusions.

### HTT levels influence lncRNA *NEAT1* levels

**Fig. 5.**
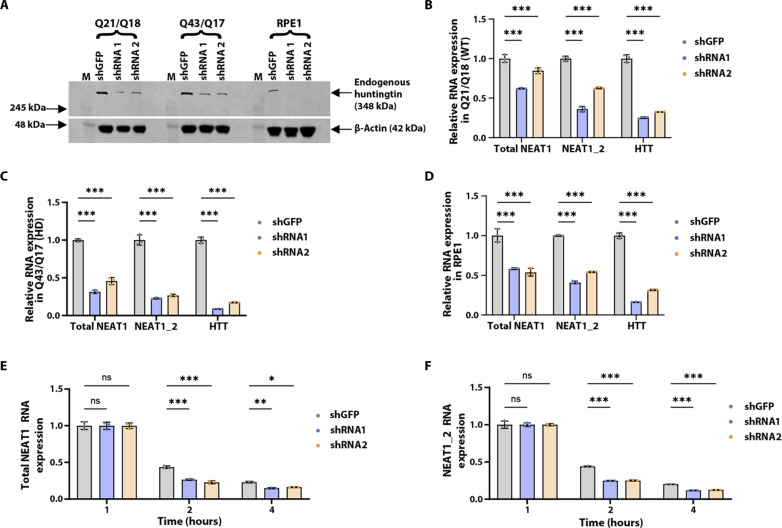
HTT knockdown reduces *NEAT1* levels and HTT stabilizes lncRNA *NEAT1*. (**A**) Western blot analysis of HTT knockdown (KD) by short hairpin RNAs (shRNAs) in WT (Q21/Q18), HD (Q43/Q17) fibroblast, and RPE1 cell lines. (**B** to **D**) RT-qPCR quantification of lncRNA *NEAT1* isoforms upon HTT KD by shRNAs in WT (Q21/Q18), HD (Q43/Q17) fibroblasts, and RPE1 cell lines, respectively. (**E** and **F**) Graphs depicting the decay of total *NEAT1* (E) and *NEAT1_2* (F) in shGFP control and shRNAs HTT KD RPE1 cells following actinomycin D treatment. U6 was used as the control gene and data were analyzed using two-way ANOVA. Data are shown as means ± SD; *n* = 3 (technical replicates). ***P < 0.001, **P < 0.01, and *P < 0.05, ns, not significant.

To understand whether there is a relationship between HTT and *NEAT1* levels, we knocked down HTT in WT (Q21/Q18) and two HD (Q43/Q17 and Q57/Q17) fibroblast cell lines using both small interfering RNAs (siRNAs; fig. S7A, C to E) and short hairpin RNAs (shRNAs; Fig. 5, A to C). HTT knockdown resulted in a reduction of both isoforms of *NEAT1* in both WT and HD fibroblasts. We also assessed *NEAT1* levels in two control cell lines: Human embryonic kidney (HEK) 293T cells expressing WT HTT and a derivative cell line null for full-length HTT (fig. S7, B and F) (*45*), as well as an human telomerase reverse transcriptase (hTERT) immortalized RPE1 cell line, which is Tet-inducible for HTT knockdown (Fig. 5, A and D). Again, we observed that *NEAT1* levels were reduced upon knockdown of HTT in RPE1 cells and in HEK293T HTT null cells compared to the parental cells, expressing normal HTT (fig. S7F). Together, these data suggest that the levels of HTT protein positively correlated with the abundance of *NEAT1* lncRNA isoforms in a variety of cell lines.

We hypothesized that HTT protein may be contributing to the stability of *NEAT1* lncRNA, thereby reducing its half-life under conditions of low HTT levels. To test this possibility, we used actinomycin D to inhibit transcription in control and HTT knockdown RPE1 cells and assessed *NEAT1* levels over time via qPCR. The abundance of both *NEAT1* isoforms decreased markedly within 2 to 4 hours with a significantly rapid reduction after HTT knockdown compared to the control cells expressing normal HTT (Fig. 5, E and F). These findings suggest that HTT plays a crucial role in maintaining the stability of *NEAT1* RNA following transcription.

### HTT and *NEAT1* colocalize in RPE1 and fibroblast cell lines

**Fig. 6.**
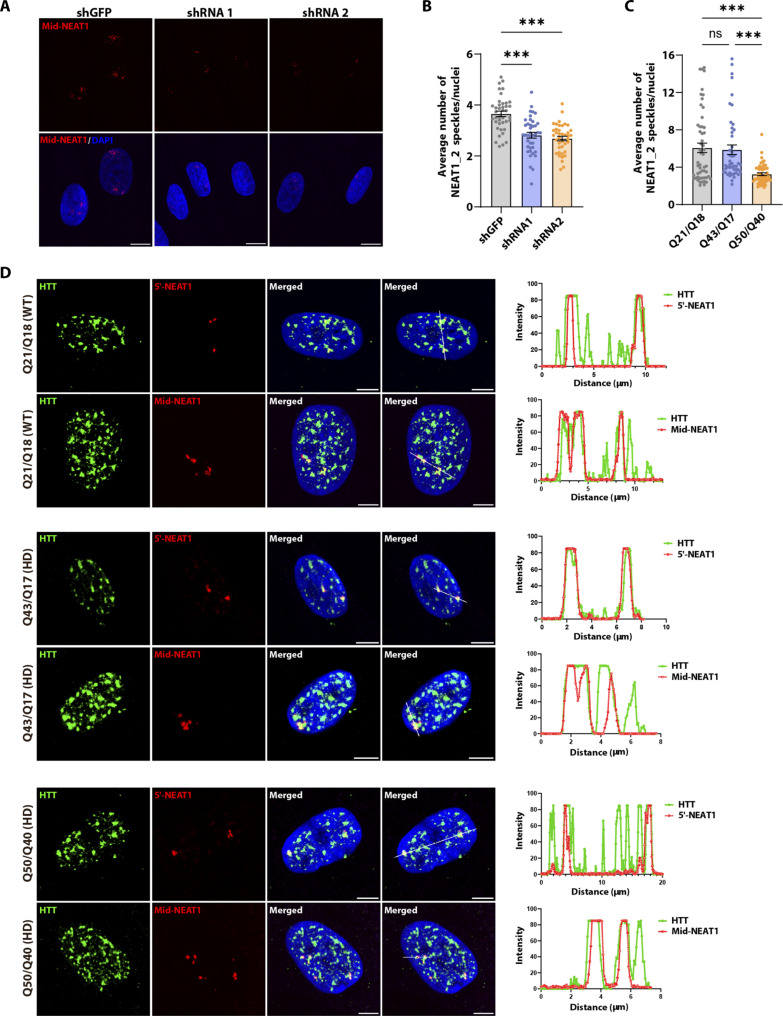
HTT colocalizes with *NEAT1*. (**A** and **B**) Representative images (A) and quantification (B) of *NEAT1_2*-positive foci in Q21/Q18 fibroblast cell lines assessed by FISH. Scale bars, 10 μm. DAPI, 4′,6-diamidino-2-phenylindole. For statistical analysis of the *NEAT1_2* RNA foci, CellProfiler software was used, and images were processed using ImageJ (Fiji app). (**C**) Quantification of *NEAT1* levels in Q21/Q18 (WT), Q43/Q17 (heterozygous HD), and Q50/Q40 (homozygous HD) fibroblast cell lines. Data represent means ± SEM. Data were analyzed by ordinary one-way ANOVA with Tukey’s test for multiple comparisons. *N* = 3 (biological replicates). ***P < 0.001. (**D**) HTT (Phospho-N17 antibody) colocalizes with both 5′-*NEAT1* and middle-*NEAT1* in WT and HD patient–derived fibroblast cells. Quantitation of HTT and *NEAT1* colocalization performed using ImageJ (Fiji app). Scale bars, 5 μm.

To further understand the relationship between HTT and *NEAT1*, we used fluorescence in situ hybridization (FISH) and confocal microscopy to visualize and quantify *NEAT1* in cells. As previously reported (*42*), we observed that both isoforms of *NEAT1* are retained in the nucleus in discrete paraspeckles (Fig. 6). Using RNA probes detecting either total *NEAT1* (5′-*NEAT1*) or only *NEAT1_2* (middle*-NEAT1*), we quantified the number of *NEAT1* paraspeckles in WT fibroblast cells (Q21/Q18) before and after HTT knockdown. The number of total *NEAT1* and *NEAT1_2* foci was significantly reduced after HTT protein knockdown in comparison to an short hairpin RNA targeting Green Fluorescent Protein (shGFP) control (Fig. 6, A and B, and fig. S8, A and B). These data suggest that HTT contributes to *NEAT1*-mediated paraspeckle formation. Moreover, the quantification of *NEAT1* foci number and intensity using FISH revealed a significant reduction in the homozygous HD fibroblast cell line (Q50/Q40) compared to the WT (Q21/Q18) and heterozygous HD (Q43/Q17) fibroblasts (Fig. 6C and fig. S8, C to E), mirroring the qPCR results (Fig. 4B), and suggesting a potential deficit in *NEAT1*-mediated paraspeckles in homozygous HD cells. Moreover, we detected changes in *NEAT1* paraspeckle morphology between WT and HD fibroblasts. Measurement of the average area of *NEAT1_2* paraspeckles (µm^2^) showed that the size of the paraspeckles was smaller in the two HD fibroblast cell lines, Q43/Q17 and Q50/Q40, compared to those in the WT cell line (Q21/Q18; fig. S8, F and G).

Our HTT-IP data suggests that HTT can bind to both isoforms of *NEAT1* (Fig. 3D). To confirm HTT-*NEAT1* interaction, we combined immunofluorescence (IF) with RNA-FISH to examine whether HTT colocalizes with both isoforms, 5′- and middle-*NEAT1* RNA within the cells. The antibody EPR5526 (Abcam) (*31*), used for RIP, shows diffuse staining of huntingtin in the cytoplasm and nucleus and does not identify speckles suitable for colocalization analysis with *NEAT1* RNA probes. Therefore, we used another antibody, termed phospho-N17 as detailed in (*10*), that recognizes HTT phosphorylated on serine residues 13 and 16. These modifications target HTT to the nucleus and thus this antibody enables nuclear HTT to be visualized by IF, revealing distinct nuclear puncta (*10*, *25*, *46*). We observed colocalization of HTT with both 5′- and middle-*NEAT1* probes in WT (Q21/Q18), heterozygous HD (Q43/Q17), and homozygous HD (Q50/Q40) fibroblasts (Fig. 6D and fig. S9A). Colocalization was also observed in RPE1 cells (fig. S9B). These results further support direct HTT-*NEAT1* interaction and suggest that HTT participates in most *NEAT1*-positive nuclear paraspeckles.

We performed Pearson’s correlation coefficient analysis on colocalization images to assess differences between WT (Q21/Q18) and HD (Q43/Q17 and Q50/Q40) fibroblast cell lines. The analysis revealed no significant distinctions between the WT and HD cell lines (fig. S9C). Subsequently, we quantified the percentage of *NEAT1* foci colocalized with phospho-N17 HTT in these cell lines, demonstrating that approximately 75 to 80% of *NEAT1* foci colocalized with HTT foci. Both HD cell lines exhibited a trend toward reduced colocalization. While statistical significance was not consistently reached for the heterozygous HD (Q43/Q17) cells, the percentage colocalization in homozygous HD (Q50/Q40) cells was significantly decreased compared to WT (fig. S9D). Again, a stronger phenotype in the HD (Q50/Q40) cells suggests a deficit in *NEAT1* paraspeckle-related functions for mHTT.

A large proportion of HTT protein also formed nuclear foci without colocalizing with *NEAT1*. HTT is also known to associate with SC35^+^ nuclear speckles (*25*), while *NEAT1* foci are reported to predominantly localize at the peripheries of SC35^+^ nuclear speckles (*47*, *48*). Therefore, we assessed if phospho-N17 HTT colocalized with both SC35^+^ nuclear speckles and *NEAT1* paraspeckles. Confirming previous studies, we observed that phospho-N17 HTT colocalized with SC35^+^ nuclear speckles, in addition to *NEAT1_2* foci in RPE1 cells (fig. S9E).

## DISCUSSION

Our study demonstrates the ability of HTT protein to directly bind to RNA species, both in vitro and in cells, suggesting its potential involvement in RNA-mediated pathways. In our HTT RIP-seq experiments, we identified a G-rich RNA motif sequence with a high affinity for HTT-HAP40, a major proteoform of HTT in cells (*29*). Our results also show that HTT interacts with *NEAT1*, which plays a crucial role as a structural scaffold for paraspeckles. Paraspeckles are dynamic, membrane-less nuclear bodies that influence various fundamental cellular functions and gene expression networks, particularly in response to stress induced by factors such as viral infections, proteasome inhibition, and mitochondrial stress (*21*, *37*, *49*). *NEAT1* harbors conserved G-quadruplex motifs (*35*), aligning with our in vitro motif analysis and assays indicating the preference of HTT for G-rich sequences.

Our study has unveiled a reduction of *NEAT1* levels in HD cell models compared to their WT counterparts. The reduction of *NEAT1* levels in HD isogenic NPCs may be attributed to decreased expression of total HTT protein, as previously observed in these cell lines with increasing polyglutamine repeats (*30*). In a previous study by Cheng *et al.* (*40*), it was reported that knockdown of mHTT in vitro and in vivo restored elevated *NEAT1* levels to the WT levels in BACHD mice. This aligns with our finding that total HTT protein knockdown reduces *NEAT1* levels, underscoring the essential role of HTT in *NEAT1*-mediated paraspeckles. Furthermore, our results indicate a decrease in *NEAT1* foci numbers, size, intensities, and percentage colocalization in HD fibroblasts, particularly in the homozygous HD cell line (Q50/Q40). Again, this decrease may be linked to the reduced levels of HTT protein in these cell lines, as previously reported (*25*). *NEAT1* binding proteins such as NONO, SFPQ (splicing factor proline and glutamine rich), and RBM14 (RNA Binding Motif Protein 14) are crucial for paraspeckle biogenesis and maintaining the stability of the lncRNA, preventing its degradation (*37*). In our study, we found that HTT also plays a role in stabilizing both short and long *NEAT1* isoforms, similarly aiding in preventing the degradation of *NEAT1*.

Recent research has highlighted the multifaceted functions of paraspeckles, including the regulation of various RNA-centric cellular processes like mRNA retention, A-to-I editing, mRNA cleavage, and protein sequestration (*43*, *49*–*51*). Our HTT RIP-seq analysis also revealed the enrichment of other transcripts such as *ADARB1*, *EIF4A1*, and *DHX58* helicases that are involved in stress response and other antiviral signaling pathways (*52*–*56*). Last, we identified HTT RNA transcripts enriched in the WT NPCs (Q30), highlighting the HTT protein's association with its own mRNA as reported previously (*17*). This enrichment was specific to the WT NPCs and not observed in other IP samples. Our analysis also unveiled the enrichment of various other lncRNAs such as *HOTAIRM1*, *RP11*, *RP1*, and *RP13*, along with mitochondrial-encoded RNAs in all immunoprecipitated samples. These transcripts are linked to mitochondrial-functional pathways and RNA regulatory mechanisms, including splicing, transcription, and translation regulation. Cross-regulation between *NEAT1*-mediated paraspeckles and mitochondria has been reported earlier, where depletion of *NEAT1* substantially affects mitochondrial dynamics and function by altering the sequestration of mito-mRNAs within paraspeckles under stress conditions (*49*). Mitochondrial dysfunction is an early pathological mechanism in HD, where mHTT disrupts mitochondria releasing mito-RNAs into the cytoplasm. This, in turn, up-regulates the innate immune response in the most vulnerable cell type, striatal spiny neurons (*4*, *5*, *57*, *58*). It is tempting to speculate that mHTT may lead to compromised paraspeckle function, resulting in a reduced ability to cope with mitochondrial stress and potentially leading to cell death in relevant HD tissues. The function and mechanisms underlying these interactions, as well as potential differences in WT and HD conditions, warrant further investigation.

## MATERIALS AND METHODS

### Recombinant HTT-HAP40 Q23 purification

The expression of HTT-HAP40 Q23 protein was performed in Sf9 insect cells, as previously described (*13*, *28*, *59*, *60*). Plasmids were transformed into DH10Bac *Escherichia coli* competent cells and plated onto LB agar plates, and then, successfully transformed white colonies were selected and grown in 3 ml of LB media supplemented with kanamycin (50 μg/ml), gentamicin (7 μg/ml), and tetracycline (10 μg/ml). Bacmids were purified using a Miniprep kit (Qiagen); but following cell lysate neutralization, the supernatants were mixed with 0.8 ml of sterile isopropanol in a fresh tube and incubated on ice for 10 min to precipitate the bacmid DNA. The DNA was pelleted by centrifugation, the supernatant was removed, and the pellets were left to air dry before resuspension in 50 μl of elution buffer from the kit.

Exponentially growing Sf9 cells were diluted to 4 × 10^5^ cells/ml in serum-free insect media and 0.5 ml of cell suspension per well was used to seed a 24-well plate to form a monolayer of cells. Plates were incubated at 27°C for 1 h. Per well seeded in the 24-well plate, 2 μl of the X-tremeGENE 9 transfection reagent was mixed with 100 μl of supplement-free insect cell growth medium and 10 μl of a solution (0.2 μg/μl) of recombinant bacmid DNA. The transfection mixture was incubated for 15 to 20 min to enable complex formation before the addition of this mixture to a seeded well in the 24-well plate. Transfection plates were incubated for 4 to 5 hours at 27°C before the addition of 1.5 ml of insect serum-free medium supplemented with 10% (v/v) of heat-inactivated fetal bovine serum (FBS) and 1% (v/v) antibiotic-antimycotic [penicillin (100 U/ml), streptomycin (100 μg/ml), and amphotericin B (0.25 μg/ml)]. Cells were incubated at 27°C incubator for 72 to 96 h before harvesting of the P1 virus stocks and the recombinant virus titer was sequentially amplified by this method to generate P3 virus stocks.

Cells at ∼4.5 million cells/ml were infected with 8 ml of P3 recombinant baculovirus per 4 liters of culture in a 5-liter reagent bottle and grown at 37°C until cell viability reached 80 to 85%, normally ~72 hours after infection. The volume of culture grown can be scaled according to the required yield of protein. For full-length HTT-HAP40, a ratio of 1:1 HTT:HAP40 P3 baculovirus was used for infection.

For purification, cells were harvested by centrifugation [JLA8.1000 (Beckman), 2500 rpm, 10°C, 10 min] and resuspended in ~40 ml of lysis buffer [20 mM Hepes (pH 7.4), 300 mM NaCl, and 2.5% (v/v) glycerol] per liter of production. Cell suspensions were lysed with two freeze-thaw cycles. Lysates were then diluted threefold in lysis buffer and clarified by centrifugation [JLA16.250 (Beckman), 14,000 rpm, 10°C, 1 hour]. The supernatant was incubated with ~1 ml of anti-FLAG resin (Sigma-Aldrich) per liter of production processed and rocked for 2 hours at 4°C. The resin was washed with ~100 CV lysis buffer, and then proteins were eluted with 5 column volumes (CV) lysis buffer supplemented with 3× FLAG peptide (250 μg/ml). Crude protein samples from FLAG-eluted fractions were then pooled, concentrated using Amicon spin filters to ~0.1 CV (molecular weight cutoff: 100,000) and purified by size exclusion chromatography using a Superose 6 Increase 10/300 GL column (Cytiva Life Sciences) in buffer containing 20 mM Hepes (pH 7.4), 300 mM NaCl, 2.5% (v/v) glycerol, and 1 mM tris(2-carboxyethyl)phosphine (TCEP). To avoid overloading the gel filtration column, only 0.5- to 1-ml sample was applied per run. Peaks corresponding to monodisperse protein were pooled, concentrated, and flash-frozen at 5 to 10 mg/ml and stored at −80°C until use. Sample purity was assessed by SDS–polyacrylamide gel electrophoresis (SDS-PAGE).

### Surface plasmon resonance

Studies were performed using a Biacore T200 (GE Health Sciences). An SA chip was primed with 3× 60-s injection with 50 mM NaOH, followed by approximately 500 response units (RU) of biotinylated nucleic acid substrate diluted in assay buffer [20 mM Hepes (pH 7.4), 50 mM KCl, 2 mM MgCl_2_, 1 mM TCEP, 2.5% glycerol (v/v), and 0.005% Tween 20 (v/v)] coupling to flow channels, with an empty flow channel used for reference subtraction. Following substrate capture, the assay buffer was flowed over the chip until a stable baseline was achieved. Protein dilutions were prepared in assay buffer and experiments were performed using multicycle kinetics with 600-s contact time, 600-s dissociation time, and flow rate (30 μl/min) at 20°C. *K*_d_ values were calculated using steady-state affinity fitting with the Biacore T200 evaluation software.

### Fluorescence polarization assay

Experiments were performed in 384-well black polypropylene PCR plates (Axygen) in 20-μl volume. In each well, 18 μl of protein solutions in assay buffer [20 mM Hepes (pH 7.4), 50 mM KCl, 2 mM MgCl_2_, 1 mM TCEP, 2.5% glycerol (v/v), and 0.005% Tween 20 (v/v)] was diluted, followed by the addition of 2 μl of 50 to 500 nM 5'-FAM–labeled nucleic acid substrate to each well. Following 1-min centrifugation at 1000 rpm, the plate was incubated for 10 min before FP measurements with a BioTek Synergy 4 (BioTek) at excitation and emission wavelengths of 485 and 528 nm, respectively. The data were processed in GraphPad Prism using Sigmoidal, 4PL, X is log(concentration) fit.

### Electrophoretic mobility shift assay

One percent agarose gel was prepared with 2 μl of ethidium bromide (BioShop Canada Inc., catalog no. ETB444.1). HTT-HAP40 Q23 (WT) protein was serially diluted from 15 to 0.2 μM in the assay buffer [20 mM Hepes (pH 7.4), 50 mM KCl, 2.5% glycerol (v/v), 2 mM MgCl_2_, 0.005% Tween 20 (v/v), and 1 mM TCEP]. The ssRNA and dsDNA oligonucleotide substrates were added to each reaction tube at a 1 μM final concentration and incubated on ice for 10 min. For *NEAT1* fragments, the concentration used was 0.25 μM. The gel was run in 0.5× tris-acetate-EDTA buffer at 100 V for 40 min for shorter oligos and 1 hour for *NEAT1* fragments. The agarose gels were imaged under a UV transilluminator.

### Cell culture

Control and HD patient–derived fibroblasts, i.e., Q40/Q18 (HD), Q43/Q19 (HD), and Q19/Q17 (WT), that were used for HTT RIP and sequencing were cultured as detailed in Gall-Duncan *et al.* (*61*). In addition, the hTERT-immortalized control (Q21/Q18) and HD patient–derived fibroblasts (Q43/Q17, Q50/Q40, and Q57/Q17) used for investigating the relationship between HTT and *NEAT1* were generously provided by R. Truant and have been previously described (*25*). These cells were cultured in Dulbecco’s modified Eagle’s medium (DMEM; Life Technologies, #10370) with 15% (v/v) FBS (Gibco, #12484-028), 1× GlutaMAX (Life Technologies, #35050), and 1× penicillin-streptomycin (PenStrep) antibiotics. The cells were grown at 37°C with 5% CO_2_. RPE1 cells (American Type Culture Collection) were cultured in 1:1 DMEM/Nutrient Mixture F-12 (DMEM/F12; Life Technologies, #11330) with 10% (v/v) FBS (Gibco, #12484-028) and 0.01% hygromycin. Cells were grown at 37°C with 5% CO_2_.

HEK293T WT and HTT null cells were unauthenticated and a kind gift from the laboratory of M. MacDonald (*45*). These cells were cultured in DMEM (Life Technologies, #10370) with 10% (v/v) FBS (Gibco, #12484-028), 1× GlutaMAX (Life Technologies, #35050), and 1× PenStrep antibiotics. Cells were grown at 37°C with 5% CO_2_.

### Isogenic NPC differentiation

hESCs were plated at a density of 30,000 to 50,000/cm^2^ per well in NPC^+^ media [equal parts DMEM/F12 (Thermo Fisher Scientific, catalog no. A4192001) and NeuroBasal medium (Thermo Fisher Scientific, catalog no. A3582901), BSA (50 μg/ml; Thermo Fisher Scientific, catalog no. B14), 1× PenStrep (Thermo Fisher Scientific, catalog no. 15140122), 1× MEM Non-Essential Amino Acids Solution (MEM NEAA) (Thermo Fisher Scientific, catalog no. 11140050), 1× N-2 (Thermo Fisher Scientific, catalog no. 17502001), 1× B-27 Supplement Minus Vitamin A (Thermo Fisher Scientific, catalog no. 12587010), human leukemia inhibitory factor (10 μg/ml; Merck Millipore, catalog no. LIF1010), 2 μM SB431542 (STEMCELL Technologies, catalog no. 72232), 3 μM CHIR99021 (STEMCELL Technologies, catalog no. 72052), and 0.1 μM Compound E (STEMCELL Technologies, catalog no. 73952)] supplemented with 10 μM Y-27632 (STEMCELL Technologies, catalog no. 72304) on Geltrex coated plates (Thermo Fisher Scientific, catalog no. A1413301). This is day 1 of differentiation. NPC^+^ medium was replaced on a daily basis till day 7. On day 7, the cells were passaged using Accutase (Thermo Fisher Scientific, catalog no. A1110501), split at a ratio of 1:10, and plated in NPC^−^ medium (NPC^+^ medium without Compound E) with 10 μM Y-27632. The cells were passaged at confluency >90%. NPCs at passage 5 or higher were then subsequently used for experiments.

### UV cross-linking

A total of 10 × 10^6^ cells were plated on 100-mm culture plates. After 24 hours, the plates were washed with 1× phosphate-buffered saline (PBS). Thereafter, 2 ml of PBS to each of the plates was added and placed on a tray of ice. The whole setup was plated inside the UV cross-linker (VWR, catalog no. 89131-484) without the lid. The plate was placed 15 cm from the light source. The cross-linker was operated at 254-nm UV light with an energy setting of 400 mJ/cm^2^ for 10 min. After cross-linking, the cells were collected with a cell scraper and centrifuged at 4°C, 300*g* for 5 min. The supernatant was discarded, and the pellets were flash-frozen in liquid nitrogen and stored at −80°C freezer.

### RNA immunoprecipitation from isogenic NPCs and fibroblasts

The pellets were retrieved from −80°C and immediately resuspended in 1 ml of cold cross-linking and immunoprecipitation (CLIP) lysis buffer [50 mM tris-HCl (pH 7.4), 100 mM NaCl, 1% NP-40, 0.1% SDS, 0.5% sodium deoxycholate, 1× protease inhibitor, and RNasin plus ribonuclease (RNase) inhibitor]. The cells were lysed on ice for 15 min and sonicated in Bioruptor at a “low” setting, 4°C for 5 min with 30 s on and 30 s off. Four units of deoxyribonuclease (DNase) were added, and the tubes were incubated in a thermomixer at 37°C with shaking at 1200 rpm for 30 min. Next, the tubes were transferred on ice, and, immediately, 50 mM final concentration of EDTA was added. The tubes were centrifuged at 20,000*g* at 4°C for 15 min to pellet debris. The supernatant was transferred to a fresh tube.

One hundred twenty-five microliters of beads for each sample was washed twice in 500 μl of cold CLIP lysis buffer, the beads were resuspended in 100 μl of cold CLIP lysis buffer, and 10 μg of EPR5526 (Abcam) anti-HTT antibody was added to the resuspended magnetic beads. The beads were rotated at 4°C for 1 to 2 hours. The tubes were centrifuged gently and placed on a magnetic rack; the supernatant was removed. The beads were further washed twice in 500 μl of cold CLIP lysis buffer. Beads for control IP (without any antibody) were prepared in the same way. Fifty microliters of cell lysates was kept for Western blot input. The remaining cell lysates were added to the prepared magnetic beads, and the tubes were kept rotating at 4°C overnight. The next day, the tubes were placed in a magnetic rack, and the cell lysate flowthrough was collected. Beads were washed further twice with 900 μl of cold high salt wash buffer [50 mM tris-HCl (pH 7.4), 500 mM NaCl, 1 mM EDTA, 1% NP-40, 0.1% SDS, and 0.5% sodium deoxycholate] and once with 500 μl of cold CLIP lysis buffer. One hundred fifty microliters of proteinase K buffer [50 mM tris-HCl (pH 7.4), 150 mM NaCl, 1 mM MgCl_2_, 0.05% NP-40, 1% sodium deoxycholate, and proteinase K (1.2 mg/ml)] was added to the beads and incubated at 55°C for 30 min while shaking to digest proteins. After this reverse cross-linking step, the tubes were gently centrifuged using a tabletop centrifuge and placed on a magnetic rack. The supernatants were transferred to new tubes, 600 μl of TRIzol reagent was added, and the tubes were incubated for 10 min at room temperature. The RNA was further purified using a Direct-zol RNA MiniPrep plus kit (Zymo Research, catalog no. R2071). The purified RNA was eluted in RNases and DNase-free water, and concentrations were estimated by nanodrop before library preparation.

### SDS-PAGE and Western blotting for IP validation

Fifty micrograms of protein lysate was used per sample. Samples were prepared using 4× NuPAGE LDS Sample Buffer (Thermo Fisher Scientific, catalog no. NP0007) and 10× NuPAGE Sample Reducing Agent (Thermo Fisher Scientific, catalog no. NP0004) and denaturing the samples at 70°C for 10 min. The denatured samples were electrophoresed at 120 V for 3 hours on NuPAGE 4–12% Bis-Tris proteins gels (Thermo Fisher Scientific, catalog no. NP0321BOX) in NuPAGE Mops SDS Running Buffer (Thermo Fisher Scientific, catalog no. NP0001). Samples were run in parallel with Full-Range Rainbow MW Markers (Thermo Fisher Scientific, catalog no. RPN800E) and/or HiMark Pre-stained Protein Standard (Thermo Fisher Scientific, catalog no. LC5699). Gels were wet-tank–transferred to polyvinyl difluoride (PVDF) Western Blotting Membranes (Sigma-Aldrich, catalog no. 3010040001; activated in 100% methanol for 1 to 2 min before use) in tris-glycine (with 10 to 20% methanol) overnight (16 to 24 hours typically) at 4°C using a constant voltage of 30 V. The next day, membranes were blocked in 5 to 10% w/v milk dissolved in 1× tris-buffered saline (TBS) + 0.1% Tween 20 (TBST) for 1 hour at room temperature. Blots are then incubated with primary antibody at room temperature for 2 hours using the same solution used for blocking, washed three times in 1× TBST at room temperature (10 min per wash), incubated with secondary antibody at room temperature for 1 hour in the same solution used for blocking, washed three times in TBST at room temperature (10 min per wash), and then detected with ECL (GE Healthcare Amersham ECL Prime Western Blotting Detection Reagent, catalog no. RPN2232) by autoradiograph. Densitometric quantification of bands is performed using Image Studio Lite Version 5.2 (LI-COR Biosciences).

For HTT IP validation by Western blot from fibroblasts:

Primary antibody: Anti-HTT Clone 1HU 4C8 (1:1000, EMDmillipore, catalog no. MAB2166).

Secondary antibody: Peroxidase-AffiniPure Sheep Anti-Mouse IgG H + L (1:2000; Cedarlane Labs, catalog no. 515035062).

For HTT IP validation by Western blot from NPCs:

Primary antibody: Anti-HTT 2B7 (2:1000; CHDI-90000830-5, Coriell ID #CH03023).

Secondary antibody: Anti-mouse IRDye 800CW (1:10,000; LI-COR Biosciences, 926-32210 or 926-32212).

### RNA library preparation

RIP-seq libraries were prepared by using SMARTer Stranded Total RNA-Seq Kit version 2 (Pico Input Mammalian; Takara, 634413) as per the manufacturer’s instructions. Subsequently, the libraries were subjected to paired-end sequencing with a read length of 150 bp on the Illumina HiSeq X Ten platform, ensuring a minimum sequencing depth of 30 million reads per sample. For both input and immunoprecipitated (IP) samples, cDNA synthesis was carried out using the High-Capacity cDNA Reverse Transcription Kit (Thermo Fisher Scientific, 4368814). qPCR was performed using the PowerUp SYBR Green Master Mix (Applied Biosystems, catalog no. A25742) on either the StepOnePlus Real-Time PCR System (Applied Biosystems) or the CFX96 Touch Real-Time PCR Detection System (Bio-Rad). qPCR validation of *NEAT1* was performed using the primers from enriched peaks, NEAT1f: ACCCTTAATGCCAGGGCTAAC for forward and NEAT1r: TGTCTTCATTTCATGCCCGC for reverse primers. To normalize the qPCR data, either U6, RPS28, or glyceraldehyde-3-phosphate dehydrogenase (GAPDH) was used as the endogenous control gene. The fold change was calculated using the ΔΔCt method.

### High-throughput sequencing data alignment and analysis

RIP-seq reads were aligned to the human reference genome hg38 by using STAR (version 2.6.1c) (*62*) with the reference annotation GENCODE version 25 (*63*). The genes bound by HTT were defined as genes enrichment fold change [log_2_(IP/INPUT)] > 1 and *P*_adj_ < 0.05. Functional enrichment analysis was performed through the web server g:Profiler (*64*) using annotated genes as background.

### MEME motif analysis and sequence annotation

For MEME motif sequence analysis, the protocol used was followed as in (*65*) with modifications. Briefly, with the resulting human genome (hg38) mapped reads as bam files, peaks were called using MACS2 with a *P* value threshold of 0.01. The input and IP reads were submitted jointly to account for background noise. The peaks were written out in BED format specifying the location of each peak in the human genome. Two thousand peaks were sampled using BEDTools (2.30.0), and flanking regions were extracted from the sampled peaks. Sequences of sampled peaks and flanking regions were retrieved, and a background file was generated using fasta-get-markov -m 0. A de novo motif file was generated using MEME (4.5.1) with options -rna -nmotifs 20 -w 20 -maxsize 1000000 -mod zoops.

### Reverse transcription qPCR

For RT-qPCR, cDNA synthesis was carried out using the High-Capacity cDNA Reverse Transcription Kit (Thermo Fisher Scientific, 4368814). qPCR was performed using the PowerUp SYBR Green Master Mix (Applied Biosystems, catalog no. A25742) on either the StepOnePlus Real-Time PCR System (Applied Biosystems) or the CFX96 Touch Real-Time PCR Detection System (Bio-Rad). To normalize the qPCR data, either U6, RPS28, or GAPDH was used as the endogenous control gene. The fold change was calculated using the ΔΔCt method.

### RNA preparation from patient brain tissues

Postmortem patient tissues were provided by the Neurological Foundation Human Brain Bank with institutional ethics approval no. 011654 (five patients with HD and five unaffected individuals; striatum, cerebellum, frontal pole) directed by R.L.M.F. and M.A.C. Tissues (stored at −80°C and kept immersed in liquid nitrogen during handling) were crushed with a frozen metal mortar and pestle partway buried in dry ice, and frozen crushed tissues were immediately transferred to a 1.4-mm acid-washed tube prefilled with zirconium beads and 300 to 1000 μl of TRIzol reagent. Smaller tissues were directly inserted into tubes without crushing. Tubes were inverted to ensure immersion of the whole tissue and were placed at room temperature for 10 min to allow the TRIzol reagent to denature and remove proteins bound to RNA. Tubes were placed on ice after 10 min and then placed in a MagNA Lyser Instrument (Roche; item #03358968001). Tubes were oscillated at 7000 OSC three times for 20 s each oscillation, with a 3-min incubation on ice between each 20-s oscillation. TRIzol was transferred to a different tube, RNA precipitated by an equal volume of 100% ethanol (EtOH) and then purified using the Direct-zol RNA purification kit using the manufacturer’s protocol, which includes in-column DNase treatment (Zymo Research, catalog no. R2071). Whole RNA was reverse-transcribed using the SuperScript IV First-Strand Synthesis System kit using the manufacturer’s protocol (Thermo Fisher Scientific, catalog no. 18091050).

### HTT knockdown using siRNAs

siRNAs for HTT knockdown were purchased from Horizon Discovery (#M-003737-02-0005), and control siRNAs were purchased from Sigma-Aldrich (#SIC001-5X1NMOL). A total of 2.5 × 10^5^ cells were seeded per 2 ml of media per well in a six-well tissue culture plate. The cells were transfected at seeding by Lipofectamine RNAiMAX Transfection Reagent (Thermo Fisher Scientific, #13778150), as per the manufacturer’s protocol. For every well in six-well plates, 150 μl of OptiMEM Medium (Gibco, #31985070) was mixed with 9 μl of RNAiMAX reagent, and 150 μl of Opti-MEM was mixed with 3 μl of 10 μM siRNA, separately. The two mixtures were then mixed to form a 300-μl mixture and incubated at room temperature for 5 min to allow the formation of siRNA-lipid complexes. The siRNA-lipid complex mixture was added to the wells at 250 μl per well for each six-well plate.

### Cell lines for HTT knockdown by shRNAs (RPE1 And fibroblasts)

HTT shRNA pNT153 and pNT154 from TRCN and negative control shGFP from H. He’s laboratory, sequences listed below, were cloned into lentiviral vector Tet-pLKO-neo (Addgene, #21916) using D. Wiederschain’s protocol (reference: The “all-in-one” system for the inducible expression of shRNA, Novartis Developmental and Molecular Pathways, Cambridge, MA, USA).

To produce lentivirus, shRNA and packaging plasmids were cotransfected into HEK293 cells using X-tremeGene HP DNA Transfection Reagent (Millipore, catalog no. 6366236001). Lentivirus was collected 48 hours later and filtered through a 0.45-μm acrodisc filter.

Tru-HD-Q21/Q18, Tru-HD-Q43/Q17, and RPE1 cell lines were infected with the virus along with polybrene (8 μg/ml) for 24 hours, and then the cells were growing in regular medium for 24 to 48 hours before screening with neomycin. For Tru-HD-Q21/Q18 and Q43/Q17, neomycin with a dose of 400 μg/ml was used; for RPE1, neomycin with a dose of 800 μg/ml was used. It took 3 weeks to obtain the stable cell lines.

pNT153: TRCN0000323037.

Hairpin sequence: 5′-CCGG-GCACTCAAGAAGGACACAATA-CTCGAG-TATTGTGTCCTTCTTGAGTGC-TTTTTG-3′.

pNT154: TRCN0000350710.

Hairpin sequence: 5′-CCGG-TGGTTCAGTTACGGGTTAATT-CTCGAG-AATTAACCCGTAACTGAACCA-TTTTTG-3′.

shGFP-hairpin sequence: 5′- CCGG-CCACATGAAGCAGCACGACTT-CTCGAGAAGTCGTGCTGCTTCATGTGG-TTTTTG-3′.

### SDS-PAGE and Western blot analysis

Fibroblast and RPE1 cells after siRNA or shRNA HTT knockdown were scraped and centrifuged at 4°C at 1500 rpm. Cell pellets were lysed in a lysis buffer [20 mM tris (pH 8.0), 150 mM NaCl, 10 mM MgCl_2_, 1 mM EDTA, 0.5% Triton X-100, 1× protease inhibitor, benzonase, and 1% SDS] for 10 min on ice and centrifuged at 14,000 rpm for 5 min. The supernatant was collected and total protein concentration was estimated using the Pierce BCA Protein Assay Kit (Thermo Fisher Scientific, #23225). Equal concentrations of total protein were loaded into a precast NuPAGE 4–12% Bis-Tris gel (Invitrogen, #NP0323BOX). Proteins were separated by SDS-PAGE in 1× Mops buffer (Life Technologies, #NP0001) for 3 hours at 120 V and electroblotted onto 0.2 μm of PVDF blotting membrane (Amersham Hybond, #GE10600021) overnight at 30 V in 1× transfer buffer (25 mM tris, 192 mM glycine, 0.375% SDS, and 10% methanol). The next day, the membrane was blocked in 5% (w/v) skimmed milk prepared in 1× PBST buffer and incubated with primary antibodies EPR5526 (2:10,000; Abcam, ab109115) or 2B7 (CHDI-90000830-5, #CH03023) and/or anti–β-actin (3:10,000; Santa Cruz Biotechnology, sc-47778) overnight at 4°C. The membranes were further washed three times for 10 min with 1× PBST and incubated with secondary antibodies (1:10 000; anti-rabbit, IRDye 800CW, LI-COR Biosciences, 926-32211 and 1:10,000; anti-mouse IRDye 800CW, LI-COR Biosciences, 926-32210 or 926-32212) in blocking buffer for 1 hour at room temperature. Membranes were washed three for 10 min with 1× PBST and imaged on a LI-COR Odyssey CLx.

### RNA-FISH and IF

Cells were grown on 18 mm round #1.5 coverglass in a 12-well glass-bottom cell culture dishes to approximately 80% confluence before fixing with 4% paraformaldehyde (PFA) solution in 1× PBS. The next day, the growth medium was removed, and the cells were washed with 1× PBS. The cells were then fixed with 4% PFA and incubated for 10 min at room temperature. The 4% PFA was removed, and the fixed cells were washed three times with 1× PBS. Stellaris FISH probes to the NEAT1 5′ region (LGC Biosearch Technologies, SMF-2036-1) and NEAT1 middle region (LGC Biosearch Technologies, SMF-2037-1), and the FISH was performed according to the manufacturer’s instructions and as in (*36*). The fixed cells were immersed in 70% EtOH for at least 1 hour at 4°C for permeabilization. After removing the 70% EtOH, wash buffer [2× saline-sodium citrate (SSC) and 10% formamide] was added and the coverslips were incubated for 5 min. Then, the 50-μl hybridization solutions [2× SSC, dextran sulfate (100 mg/ml), and 10% formamide] containing Stellaris NEAT1 probes (final concentration: 125 nM) were dropped onto the coverslips in the humidified chamber and were incubated for 16 hours in the dark. The coverslips were washed with wash buffer at 37°C in the dark for 30 min and with 2× SSC at room temperature for 5 min. For just FISH, the coverslips were mounted with VECTASHIELD Hard Set Mounting Medium with 4′,6-diamidino-2-phenylindole (DAPI; Vector).

The coverslips were subsequently washed with 1× PBS and incubated with blocking solution (Intercept blocking buffer, LI-COR, #927-70001, and 1× PBST) for blocking at room temperature for 1 hour. Then, the coverslips were incubated with HTT primary antibodies (phospho-N17, 1:250) in blocking solution at room temperature for 1 hour, washed three times with 1× PBS for 5 min, incubated with secondary antibody anti-rabbit (Alexa Fluor 488) in blocking solution at room temperature for 30 min, and washed three times with 1× PBS for 5 min. The coverslips were mounted with VECTASHIELD Hard Set Mounting Medium with DAPI (Vector). Confocal images were acquired using a confocal microscope (Leica SP8).

### RNA stability assay

For the RNA stability assay, the protocol was followed as in (*66*) with modifications. A total of 1 × 10^5^ RPE1 cells containing shGFP and HTT shRNA plasmids were seeded per well in a six-well plate for five time points (*t* = 0 to *t* = 4 hours). The plate was incubated overnight at 37°C, and doxycycline (2 μg/ml) was added to each well on the next day to initiate HTT knockdown. On the fifth day, cells from *t* = 0 time point were collected, spun at 470*g* for 3 min at 20°C, and frozen at −80°C. To the remaining wells, 30 μl of actinomycin D (1 mg/ml) was added to obtain a final concentration of 10 μg/ml in 3 ml of culture media. The samples were collected at 1-, 2- and 4-hour time points and the pellet was frozen at −80°C until use. The RNA was isolated using the Qiagen RNA Miniprep kit and RT-qPCR was performed. The fold change was calculated using the ΔΔCt method and all samples were normalized to the control *t* = 0 time point.

### Image processing and NEAT1 paraspeckle count

The *z*-stack images were acquired using a Leica TCS SP8 confocal microscope (Leica DMi8 CS inverted stand) with 405-, 552-, and 488-nm lasers. The images were processed and analyzed in LASX office software (version 1.4.1) and ImageJ. The *NEAT1* paraspeckles number, size, and intensities were quantified in more than 150 to 200 cells over three trials using an open-source speckle counting and area pipeline in CellProfiler (version 4.2.6).

### Data visualization and analysis

Data analysis and visualization were conducted using R (version 4.0.0), and the packages used are the following: ggplot2_3.4.1, clusterProfiler_3.16.1, ComplexUpset_1.3.3 and venn_1.11, and GraphPad Prism (version 10.1.1). The outcomes of all statistical tests including *P* values and number of samples are included in the figure panels or the corresponding figure legends. Significance was defined as any statistical outcome that resulted in a *P* value of less than 0.05 unless otherwise indicated.
