# Supplementary material for: Huntingtin is an RNA binding protein and participates in NEAT1-mediated paraspeckles
Source: Sci Adv. 2024 Jul 19;10(29):eado5264. doi: 10.1126/sciadv.ado5264 (PMC11259171; doi:10.1126/sciadv.ado5264)
Supplement: Supplementary file 1 — Figs. S1 to S9 Tables S1 and S2 Legend for data S1 [file sciadv.ado5264_sm.pdf]

Science Advances
AAAS

Supplementary Materials for

Huntingtin is an RNA binding protein and participates in *NEAT1*-mediated paraspeckles

Manisha Yadav *et al.*

Corresponding author: Cheryl H. Arrowsmith, cheryl.arrowsmith@uhn.ca

Sci. Adv. **10**, eado5264 (2024)
DOI: 10.1126/sciadv.ado5264

The PDF file includes:

Figs. S1 to S9
Tables S1 and S2
Legend for data S1

Other Supplementary Material for this manuscript includes the following:

Data S1

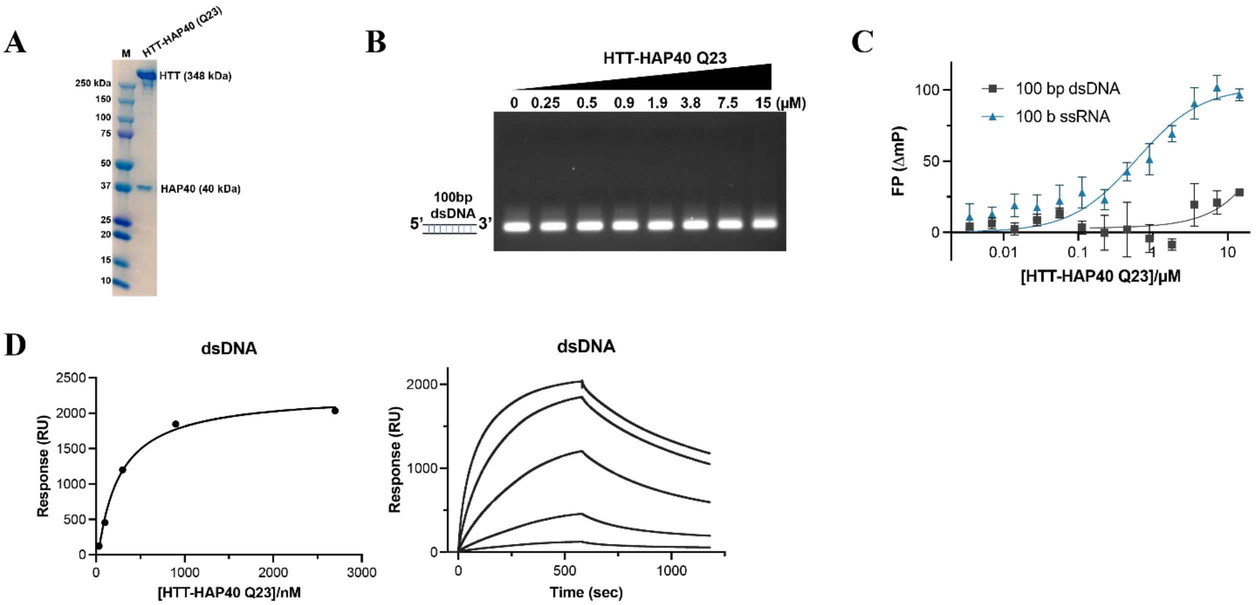

Fig. S1. HTT-HAP40 prefers to bind RNA and prefers G-rich sequences. (A) SDS-PAGE analysis showing recombinantly purified HTT-HAP40 Q23 protein after gel filtration chromatography purification. **(B)** Representative EMSA image of increasing HTT-HAP40 Q23 protein (0–15 μM) binding with 1 μM of 100bp dsDNA. DNA is in black. EMSA, electrophoretic mobility shift assay. **(C)** Representative FP binding curve of HTT-HAP40 Q23 and 100-mer random ssRNA (K_D = 1 ± 0.6 μM), dsDNA (K_D = NC). NC, not calculated, outside of range of protein concentrations tested. **(D)** Representative SPR binding curve and sensorgram of HTT-HAP40 Q23 and 100bp dsDNA (K_D = 2.0 ± 0.2 μM).

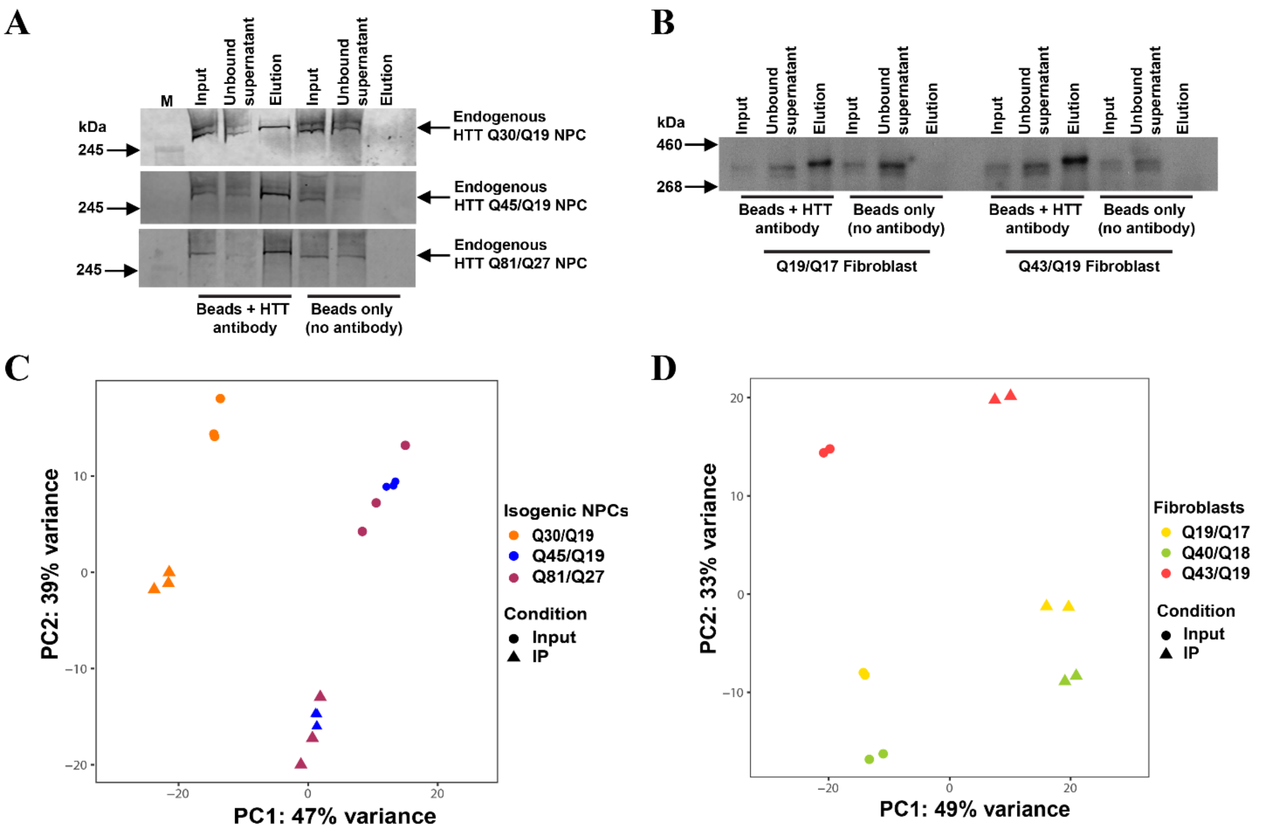

Fig. S2. HTT RIP-seq analysis in isogenic NPCs and fibroblasts. (A-B) Validation of HTT IP protocol through western blot analysis in NPCs (A) and fibroblast cell lines (B). **(C-D)** Principal components analysis (PCA) of RIP-seq dataset of WT and HD isogenic NPCs (C) in triplicates, and fibroblast (D) cell lines in duplicates.

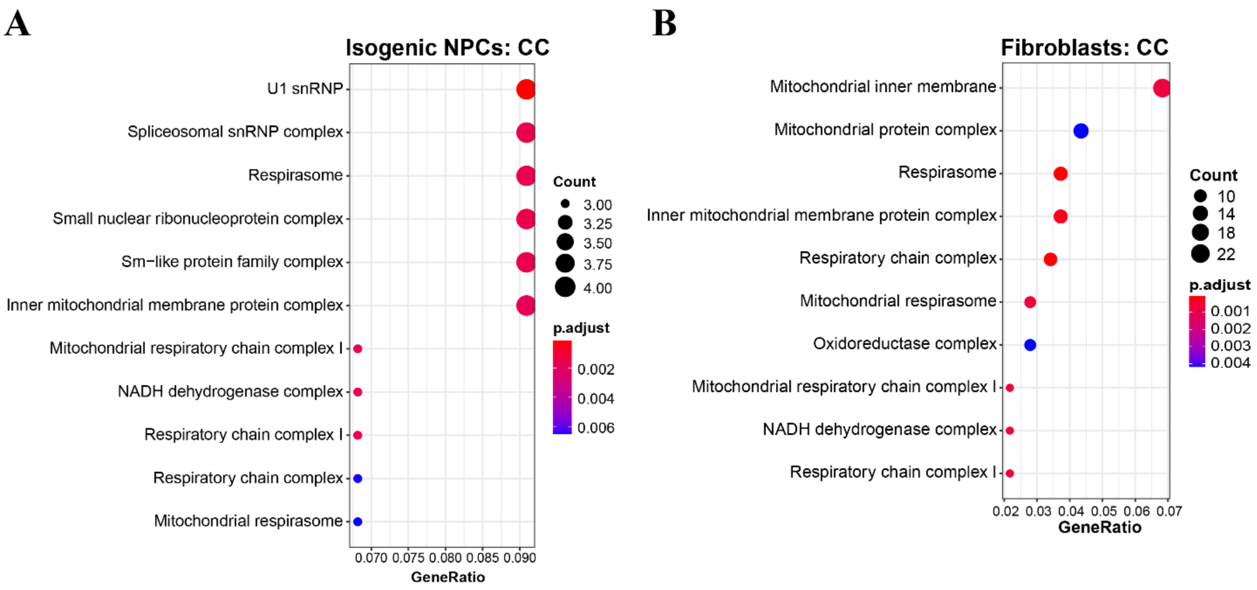

Fig. S3. HTT RIP-seq analysis in isogenic NPCs and fibroblasts. (A-B) GO enrichment analysis for cellular component (CC) in WT and expanded NPCs (A) and fibroblasts (B).

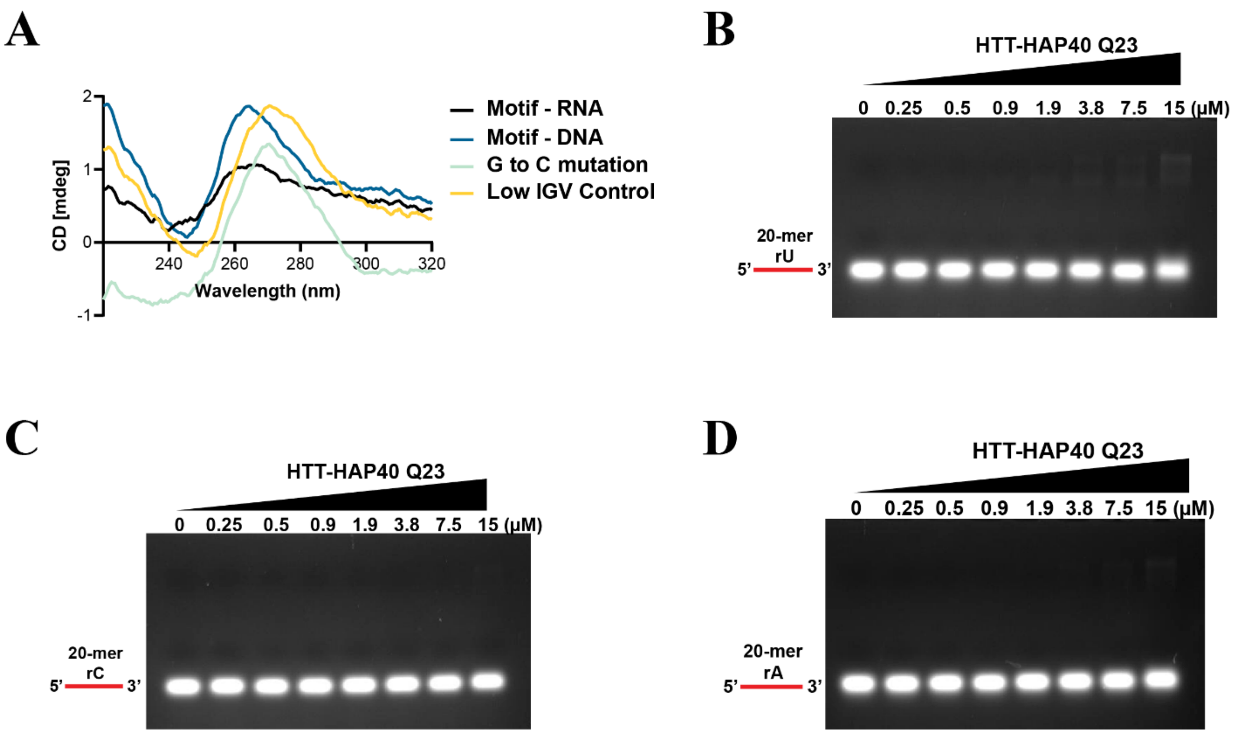

Fig. S4. (A) Circular Dichroism (CD) spectroscopy analysis for secondary structure folding of identified RNA motif (Motif-RNA), RNA to DNA motif (Motif-DNA), G to C mutated RNA, and low IGV RNA. The CD spectral features of G4 topologies exhibit peak wavelengths of ≈ 264 nm at maximum and ≈ 245 nm at minimum (*34*). **(B-D)** Representative EMSA images of increasing HTT-HAP40 Q23 protein (0–15 µM) binding with 1 µM of indicated 20-mer rU (B), rC (C), and rA (D) substrates. RNA is in red. EMSA, electrophoretic mobility shift assay.

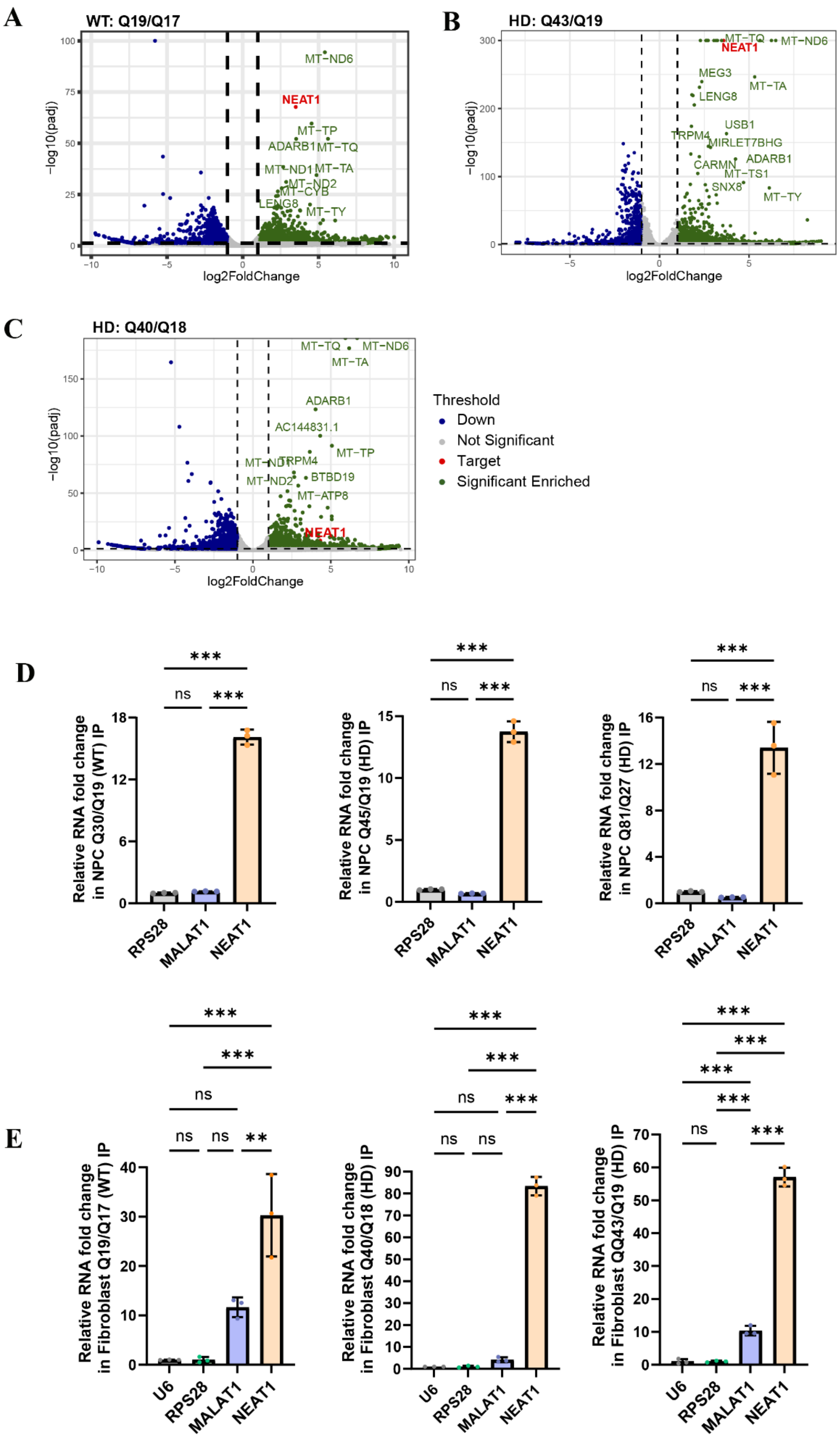
A
WT: Q19/Q17
MT−ND6
NEAT1
MT−TP
ADARB1
MT−TQ
MT−ND1
MT−TA
MT−ND2
MT−CYB
LENG8
MT−TY
−log10(padj)
log2FoldChange
B
HD: Q43/Q19
MT−TQ
MT−ND6
NEAT1
MEG3
MT−TA
LENG8
USB1
TRPM4
MIRLET7BHG
ADARB1
CARMN
MT−TS1
SNX8
MT−TY
−log10(padj)
log2FoldChange
C
HD: Q40/Q18
MT−TQ
MT−ND6
MT−TA
ADARB1
AC144831.1
MT−TP
MT−ND1
TRPM4
MT−ND2
BTBD19
MT−ATP8
NEAT1
−log10(padj)
log2FoldChange
Threshold
Down
Not Significant
Target
Significant Enriched
D
Relative RNA fold change in NPC Q30/Q19 (WT) IP
Relative RNA fold change in NPC Q45/Q19 (HD) IP
Relative RNA fold change in NPC Q81/Q27 (HD) IP
RPS28
MALAT1
NEAT1
ns
***
E
Relative RNA fold change in Fibroblast Q19/Q17 (WT) IP
Relative RNA fold change in Fibroblast Q40/Q18 (HD) IP
Relative RNA fold change in Fibroblast QQ43/Q19 (HD) IP
U6
RPS28
MALAT1
NEAT1
ns
**
***

Fig. S5. LncRNA NEAT1 enrichment validation using RT-qPCR. (A-C) NEAT1 is significantly enriched in the WT and HD IP samples derived from fibroblast cells (Log_2 fold change cutoff: 1; p-value cutoff: 0.05). **(D-E)** Both form-specific (total NEAT1) and long form-specific (NEAT1_2) enrichment evaluation by RT-qPCR from WT and expanded isogenic NPCs (D) and fibroblasts (E). Three abundant coding and noncoding transcripts (RPS28, U6 and MALAT1) are shown as negative controls. Data was analyzed using one-way ANOVA. Data are shown as mean ± s.d.; $n = 3$ (technical replicates). ***$P < 0.001$, **$P < 0.01$, ns, not significant.

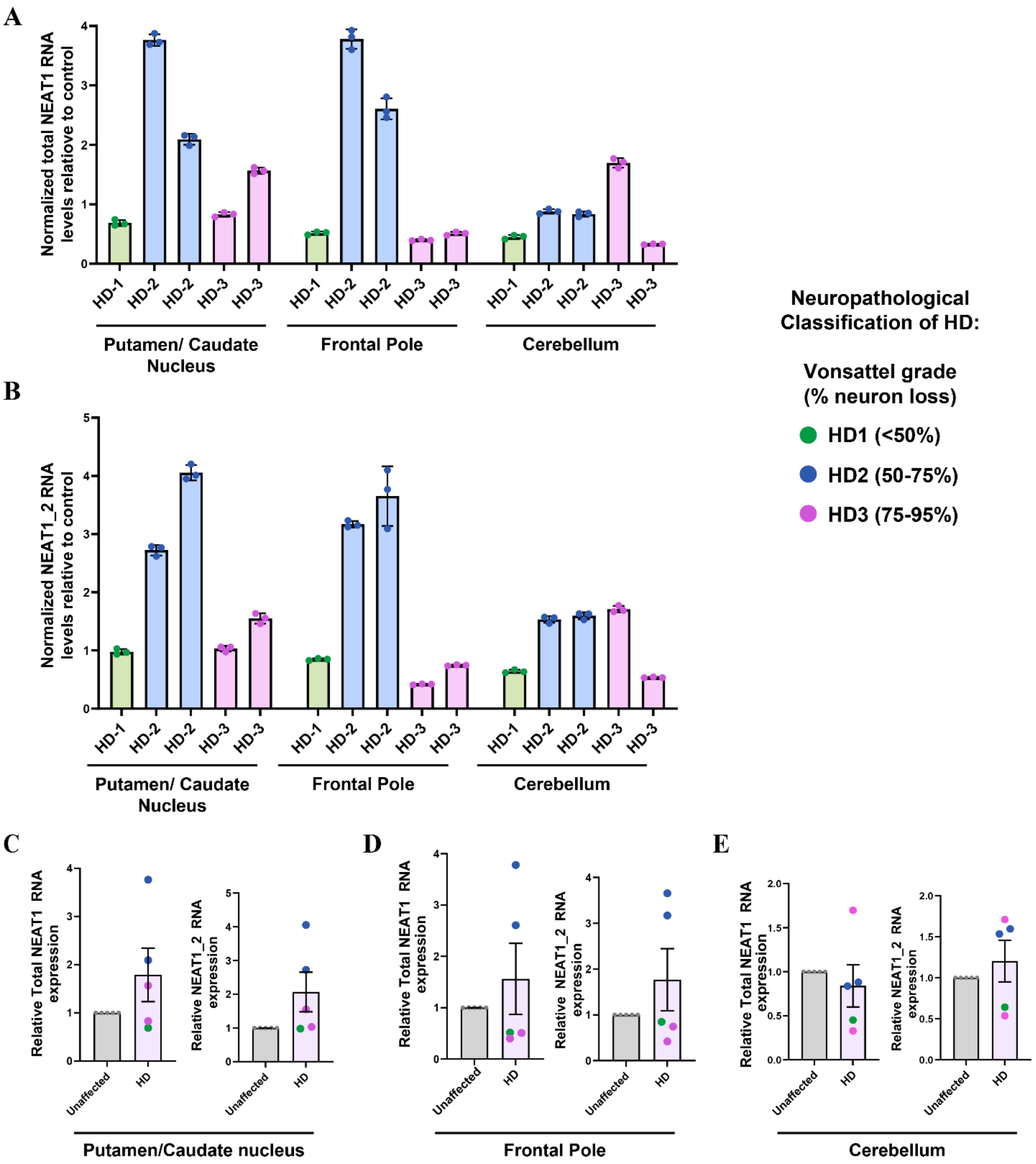

Fig. S6. Altered lncRNA NEAT1 levels in HD patient brain tissues (A-B) Total NEAT1 (A) and long NEAT1 (NEAT1_2) (B) expression levels in putamen, frontal pole, and cerebellum regions of human HD patient brain at different HD-grades. The HD patient tissues were normalized to age and sex matched unaffected brain tissues for each region, N = 5 HD (i.e. 1 individual from stage-1 HD, 2 individuals from stage-2 and 2 individuals from stage-3, total=5 individuals) and N=5 unaffected individuals/group/tissue, dots on bars represent 3 technical replicates/person. Green, blue and purple color of the dots indicate striatal neuropathological grade (HD 1–3), gray

dots indicate unaffected controls. **(C-E)** Both isoform specific NEAT1 (total NEAT1) and NEAT1_2 expression levels in putamen, frontal pole, and cerebellum regions of human HD patient brain compared to age and sex matched unaffected brain tissues (gray) for each region. N = 5 HD and N = 5 unaffected individuals/group/tissue, average of 3 technical replicates/person. The color of dots indicates striatal neuropathological grade (HD 1–3). U6 was used as a control gene. No statistics applied due to limited patient sample size.

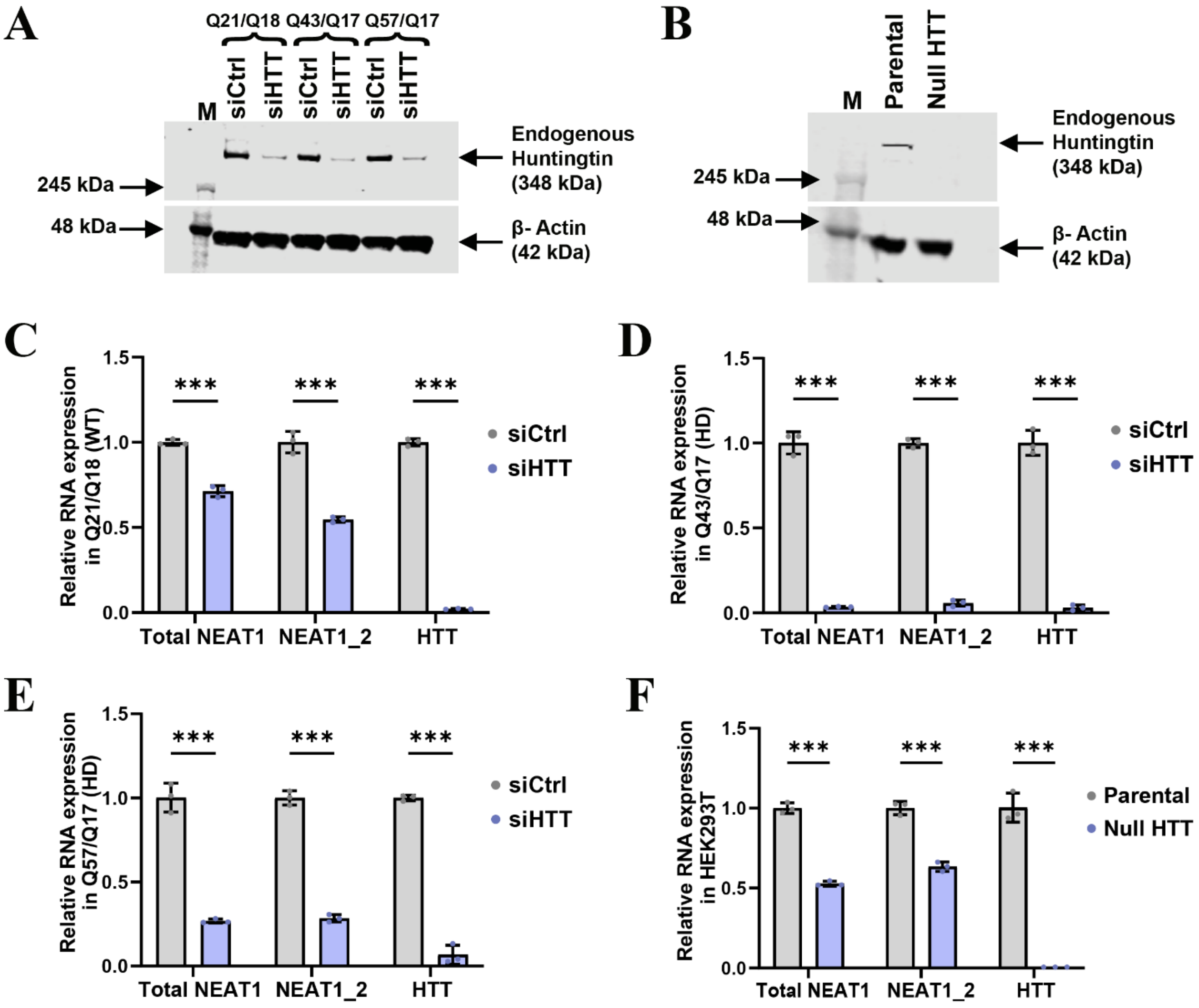

Fig. S7. HTT knockdown reduces lncRNA NEAT1 levels. (A) Western blot analysis of HTT protein knockdown by siRNAs in WT (Q21/Q18), HD (Q43/Q17) and HD (Q57/Q17) fibroblast cell lines. **(B)** Western blot analysis of HTT expression in parental and HTT knockout HEK293T cells. **(C-E)** RT-qPCR quantification of levels of lncRNA NEAT1 isoforms upon HTT knockdown by siRNAs in WT (Q21/Q18), HD (Q43/Q17) and HD (Q57/Q17) fibroblasts, respectively. **(F)** RT-qPCR quantification of levels of lncRNA NEAT1 isoforms in HEK293T parental and Null HTT cell lines. U6 was used as control gene and data were analyzed using 2-way ANOVA. Data are shown as mean ± s.d.; *n* = 3 (three technical replicates). ***P < 0.001.

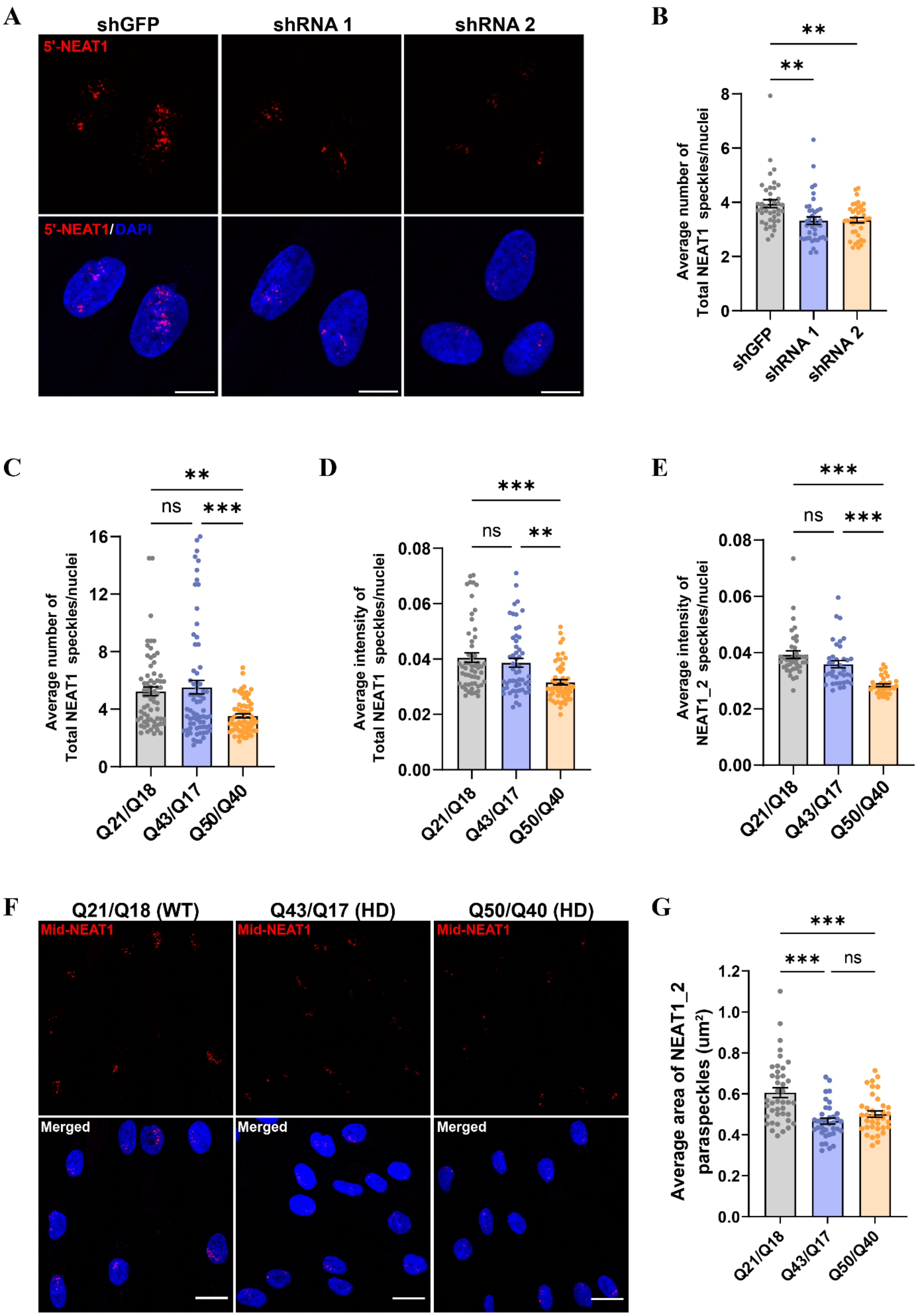

A
shGFP
shRNA 1
shRNA 2
5'-NEAT1
5'-NEAT1/DAPI
B
Average number of Total NEAT1 speckles/nuclei
shGFP
shRNA 1
shRNA 2
C
Average number of Total NEAT1 speckles/nuclei
D
Average intensity of Total NEAT1 speckles/nuclei
E
Average intensity of NEAT1_2 speckles/nuclei
Q21/Q18
Q43/Q17
Q50/Q40
ns
F
Q21/Q18 (WT)
Q43/Q17 (HD)
Q50/Q40 (HD)
Mid-NEAT1
Merged
G
Average area of NEAT1_2 paraspeckles (um²)

Fig. S8. NEAT1 lncRNA levels reduce after HTT knockdown (A-B) Representative images **(A)** and quantification **(B)** of Total NEAT1 positive foci in Q21Q18 fibroblast cell lines after HTT knockdown, assessed by FISH. Scale bar = 10 µm. For statistical analysis of the Total NEAT1 RNA foci, CellProfiler software was used, and images were processed using ImageJ (Fiji app). Data represent mean ± sem. Data were analyzed by ordinary one-way ANOVA with Tukey's test for multiple comparisons. ∗∗∗P < 0.001. **(C)** Quantification of average number of Total NEAT1 foci in WT and HD fibroblast cell lines. **(D-E)** Quantification of average intensity of Total NEAT1 and NEAT1_2 foci in WT and HD fibroblast cell lines. **(F-G)** Representative images (F) and quantification (G) of NEAT1_2 foci's average area (µm^2) in WT and HD fibroblasts. Scale bar = 20 µm. Data represents mean ± sem. Data were analyzed by ordinary one-way ANOVA with multiple comparisons. N=3 (biological replicates). ∗∗∗P < 0.001, ∗∗P < 0.01 and ns, not significant.

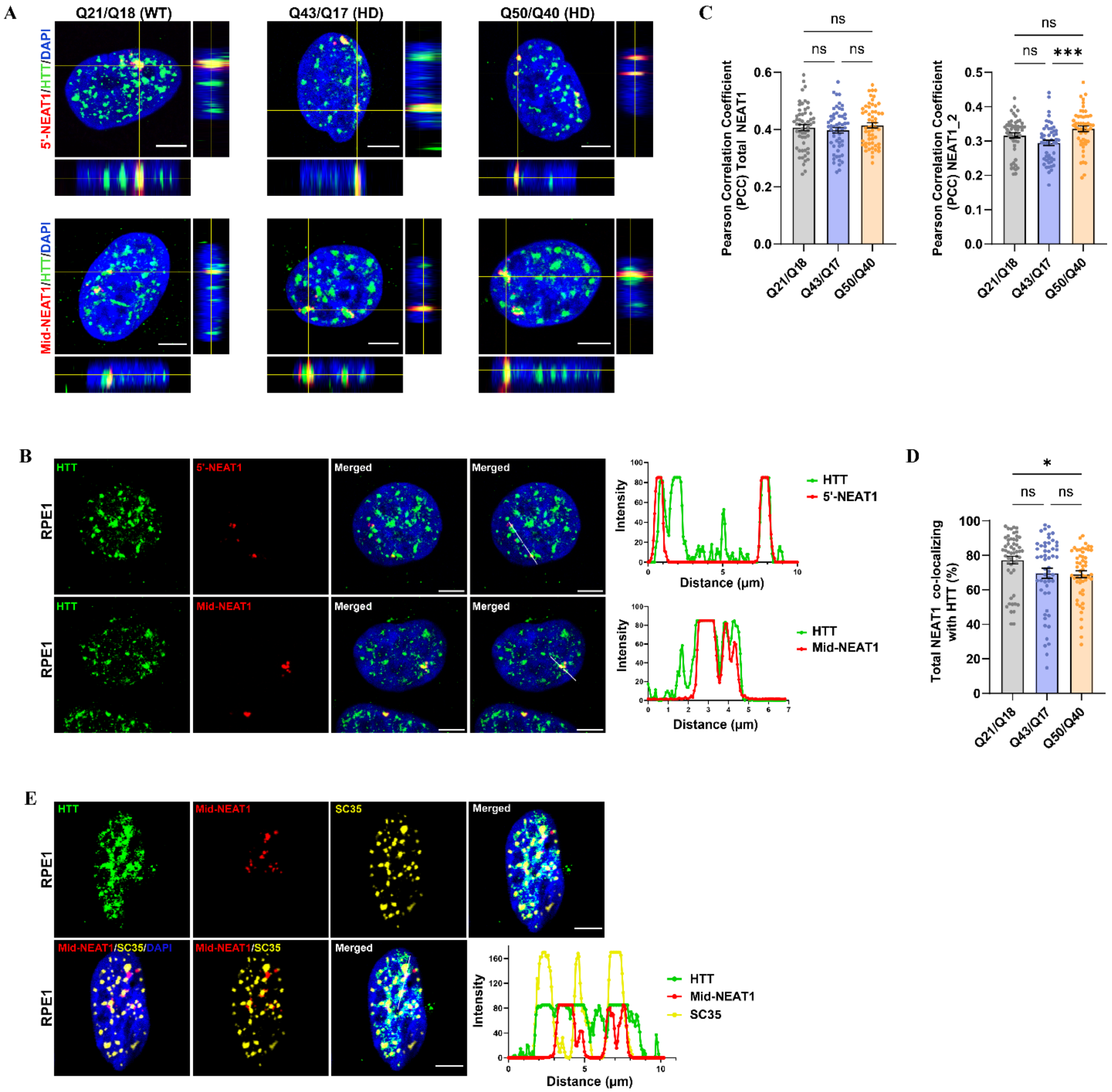

Fig. S9. NEAT1 lncRNA and HTT protein co-localization (A) Co-localization of HTT (green) and NEAT1 (Red), for all sets of orthogonal view images for WT and HD fibroblasts, the large image shows the x–y view, the bottom image shows the z–x and the right image shows the z–y. Scale bar indicates 5µm. **(B)** Confocal images showing co-localization of HTT (green) and NEAT1 (Red) in RPE1 cells. Quantitation of HTT and NEAT1 co-localization (performed using ImageJ). Scale bar indicates 5µm. **(C)** The graph shows Pearson's correlation coefficient for HTT and NEAT1 co-localization analysis in WT and HD fibroblasts. JACoP in ImageJ were used, and images were processed using ImageJ. Data represents mean ± sem; N=3 (biological replicates). **(D)** The percentage of total NEAT1 co-localized with HTT in WT and HD fibroblasts was quantified using the JACoP in ImageJ. Data represents mean ± sem. Data were analyzed by ordinary one-way ANOVA with multiple comparisons. $***P < 0.001$ and $*P < 0.05$, ns, not significant. **(E)** Co-localization of HTT (green) with NEAT1_2 (red) and SC35+ nuclear speckles

(yellow) in RPE1 cells. Quantitation of HTT, NEAT1 and SC35+ nuclear speckles co-localization (performed using ImageJ). Scale bar indicates 5μm.

Table S1. List of RNA and DNA substrates used in this study.

No.	Substrate Name	Nucleotide Sequence (5'→3')
1.	100-mer random ssRNA	RNA 100-mer: CACGUAUGAGAAGGUAUUUGCCCGAUAAUCA AUACCCCAGGCUUCUAACUUUUUCCACTCGCU UGAGCCGGCUAGGCCUUUCUGCCCGAAGUUU CGAUGG
2.	100bp random dsDNA	DNA 100-mer: CACGTATGAGAAGGTATTTGCCCGATAATCAA TACCCCAGGCTTCTAACTTTTTCCACTCGCTTG AGCCGGCTAGGCCTTTCTGCCCGAAGTTTCGAT GG<br>DNA 100-mer comp: CCATCGAAACTTCGGGCAGAAAGGCCTAGCCG GCTCAAGCGAGTGGAAAAAGTTAGAAGCCTGG GGTATTGATTATCGGGCAAATACCTTCTCATAC GTG
3.	20-mer rA RNA + 5' FAM	5' FAM - AAAAAAAAAA AAAAAAAAAA
4.	20-mer rC RNA + 5' FAM	5' FAM - CCCCCCCCCC CCCCCCCCCC
5.	20-mer rG RNA + 5' FAM	5' FAM - GGGGGGGGGG GGGGGGGGGG
6.	20-mer rU RNA + 5' FAM	5' FAM - UUUUUUUUUU UUUUUUUUUU
7.	25-mer Motif-RNA	5' FAM-AAUGGAAGGCGAGGCAGGCGGGCGU
8.	25-mer Motif-DNA	5' FAM-AATGGAAGGCGAGGCAGGCGGGCGT
9.	25-mer G to C Mutation RNA	5' FAM-AAUGCAAGCCGAGCCAGCCGCGCGU
10.	25-mer Low IGV control RNA	5' FAM-AGTTTTGAAATAGTCTAATTTATCT

Table S2. Demographic characteristics of human postmortem brain tissue.

Subject	Condition	Age (years)	Sex (M/F)	CAG	Region sampled	COD	PMI (hr)
**HD**							
1.	HD-1	32	M	17/47	Put/CN, CB, FP	Submandibular squamous cell carcinoma	14
2.	HD-2	65	M	17/43	Put/CN, CB, FP	Renal failure	14
3.	HD-2	70	F	17/42	Put/CN, CB, FP	HD	8
4.	HD-3	64	M	27/42	Put, CB, FP	Pulmonary thromboembolus	18
5.	HD-3	63	M	22/43	Put/CN, CB, FP	Pulmonary embolism	16
**Control**							
1.	Control	22	M	11/23	Put, CB, FP	Asphyxia	21
2.	Control	60	M	10/17	Put, CB, FP	Ischaemic heart disease	17
3.	Control	78	F	18/19	Put/CN, CB, FP	Aortic aneurysm	20
4.	Control	64	M	13/15	Put, CB, FP	Ischemic Heart Disease	15.5
5.	Control	68	M	17/19	Put, CB, FP	Coronary atherosclerosis	22.5

HD Huntington's disease, *COD* cause of death, *PMI* postmortem interval, *Put* putamen, *CN* caudate nucleus, *CB* cerebellum, *FP* frontal pole

Data S1. (separate file)

List of transcripts enriched in HTT RIP-sequencing for both WT and HD isogenic NPCs and fibroblasts used in this study.
